# Identification of novel flavin-dependent monooxygenase from *Strobilanthes Cusia* reveals molecular basis of indoles’ biosynthetic logic

**DOI:** 10.1186/s12870-023-04557-5

**Published:** 2023-10-31

**Authors:** Chang Liu, Mengya Cheng, Chao Ma, Junfeng Chen, Hexin Tan

**Affiliations:** 1 Department Chinese Medicine Authentication, College of Pharmacy, Naval Medical University (Second Military Medical University), Shanghai, China; 2 Department of Pharmacy, Shanghai Fourth People’s Hospital Affiliated to Tongji University School of Medicine, Shanghai, China; 3 School of Health Science and Engineering, University of Shanghai for Science and Technology, Shanghai, China; 4 Department of Vascular Disease, Shanghai TCM-Integrated Hospital, Shanghai University of Traditional Chinese Medicine, Shanghai, China; 5 Shanghai Key Laboratory for Pharmaceutical Metabolite Research, Shanghai, China

**Keywords:** *Strobilanthes Cusia*, Indoles, Biosynthesis, ScFMO

## Abstract

**Background:**

*Strobilanthes cusia* (Nees) Kuntze is a traditional medical plant distributed widely in south China. The indole compounds that originated from the plant are responsible for its pharmacological activities. However, the reason why indole ingredients are accumulated in this herb and how it is biosynthesized has remained largely unknown.

**Results:**

In this study, metabolic and transcriptional profiling measurement experiments of different *S. cusia* organs were carried out to understand the underlying molecular basis of indoles’ biosynthetic logic. A metabolic investigation demonstrated that the indoles are primarily accumulated mainly in aerial parts, particularly in leaves. RNA-seq was employed to reveal the organ specific accumulation of indoles in different *S. cusia* organs. Meanwhile, a flavin-dependent monooxygenase gene (*ScFMO1*) was found in *S. cusia*, and it has capacity to produce indoxyl from indole by the fermentation assay. Finally, we assessed the outcomes of transient expression experiment in tobacco and confirmed that ScFMO1 localizes in cytoplasm.

**Conclusions:**

Our results suggest that ScFMO1 plays a key role in biosynthesis of indoles (Indigo, indirubin, indican, etc.), it will be useful for illuminating the molecular basis of the medicinal indoles’ biosynthesis and developing strategies for improving their yields.

**Supplementary Information:**

The online version contains supplementary material available at 10.1186/s12870-023-04557-5.

## Background

*Strobilanthes cusia* is a perennial herb plant and widely distributed in south China. It has important economic value as a traditional Chinese medicine with thousands of years of medicinal history. The leaf and stem extract of *S. cusia* is one of the sources of Qingdai, which was first recorded in Kaibao Bencao in the Tang dynasty, and Nanbanlangen, the rhizome et radix of *S. cusia*, was first recorded in Sheng Nong’s herbal classic for medicinal purposes. Nowadays, they are still officially included in Chinese pharmacopoeia [1]. From *S. cusia*, 69 active substances have been discovered [2]. Indole alkaloids, one of these components, have the strongest pharmacological potential [3, 4], and are what causes the leukocyte-inhibition, anti-inflammatory, and antiviral effects [5–8]. However, most of these compounds are presented in *S. cusia* at a low content, which makes it necessary to increase the yields of these pharmacologically valuable metabolites for the growing social needs.

A potent technique for enhancing the synthesis of important chemicals in plants is the development of bioengineering methods based on metabolic regulation. The use of bioengineering technology has significantly enhanced the concentration of important compounds in many therapeutic plants, including *Catharanthus roseus* [9], *Hyoscyamus niger* [10, 11], and *Salvia miltiorrhiza* [12–14]. This is due to the discovery of the biosynthetic routes of natural products. The metabolic engineering of *S. cusia* is currently not making much progress, mostly because it is unclear how plants produce indigo. Therefore, it is crucial to fully comprehend the plant’s biosynthetic process for producing indigo.

Indoxyl, which is assumed to be biosynthesized from indole, was thought to be the source of indigo’s biosynthetic precursors. One might reasonably hypothesize that this reaction is catalyzed by a group of oxidizing enzymes. It is known that plants include a range of oxidases, including cytochrome P450s (CYPs) and flavin-dependent monooxygenases (FMOs), which are involved in a variety of metabolic pathways, including secondary metabolisms. Some endoplasmic reticulum related FMOs and CYPs catalyze the hydroxylation of secondary metabolites, and most of which are derived from mammals or microorganisms [15–17]. Recently, Inoue, Morita & Minami (2021) have reported that PtFMO from *P. tinctorium* can catalyze indole hydroxylation [18]. According to these reports, the potential enzymes catalyze the production of indoxyl from indole may also be members of the FMO family in plants. With the advancement of *S. cusia* genome and transcriptome, a lot of related candidate genes involved in the biosynthesis of indigo were revealed, but the main enzyme genes that catalyze indole to indoxyl in *S. cusia* have not been confirmed [19–22].

The aim of the research was to establish a candidate gene pool of *S. cusia* to assist in the discovery of new genes related to the indoles biosynthesis pathways. Metabolite analysis was carried out following the indications offered by the transcriptome. The biosynthesis of indole alkaloids probably occurs in the aerial part of *S. cusia* (stem and leaves). We screened out a FMO gene that was highly expressed in the leaves of *S. cusia* (*ScFMO1*) which is proved to have the capacity to produce indoxyl from indole. Meanwhile, integrated analysis of the transcriptome and the secondary metabolites will lead to an in-depth knowledge of both the pool of metabolites and biosynthetic processes for the formation of the active compounds in *S. cusia*.

## Results

### Accumulation of indoles in different *S. cusia* organs

**Fig. 1:**
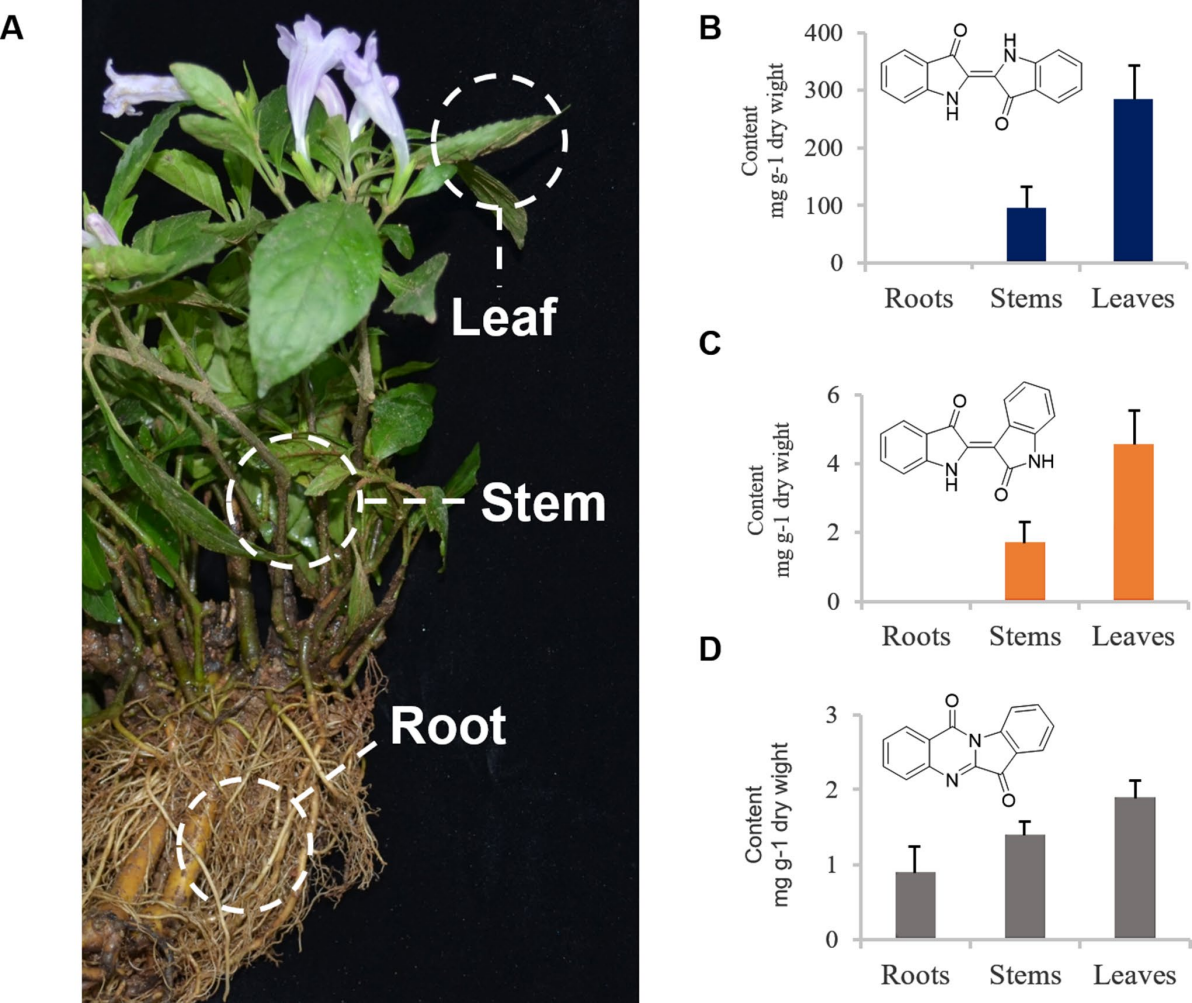
Accumulation of indigo and indirubin in different *S. cusia* organs. Mature plant of *S. cusia* **(A)**. Accumulation patterns of indigo **(B)**, indirubin **(C)**, and tryptanthrin **(D)** in different *S. cusia* organs. Bars in blue indicated content level of indigo, and orange bars indicated that of indirubin. ND, not detected

To study the accumulation pattern of indigo and indirubin in *S. cusia*, the LC-MS method was employed to detect indigo, indirubin, and tryptanthrine in roots, stems, and leaves of mature *S. cusia* (Fig. 1A). Indigo was detected to accumulate only in aerial part of *S. cusia*, with the highest content (288.72 mg/g DW) in leaves and lower (99.675 mg/g DW) in stems (Fig. 1B). A similar accumulation of indirubin level was detected in leaves (4.625 mg/g DW), and stems (1.775 mg/g DW) of *S. cusia* and undetectable in roots (Fig. 1C). The result was consistent with the fact that leaf is the effective part of this herb. On the other hand, tryptanthrine did not show obviously different accumulation in the three organs (0.925 mg/g DW in roots, 1.425 mg/g DW in stems, and 1.925 mg/g DW in leaves) (Fig. 1D).

### cDNA sequence generation, de novo assembly

To obtain a complete profile of the *S. cusia* transcriptome in different organs, nine cDNA libraries were built for samples of different organs (leaves, stems, and roots). 75,819,952 to 122,713,724 raw reads were generated from each library (Table 1). 68,347,296 to 109,571,128 clean reads in average length from 122.92 to 123.46 bp were remained for assembly after removing low quality reads. Q20 percentages (sequencing error rate < 1%) and GC percentages obtained were 99.20–99.27% and 45.71–46.46%. These results indicate that the above data have eligible quality for accurate sequence assembly and adequate transcriptome coverage. 293,666 unigenes were obtained by Trinity sequencing. The mean length of each library was 650.65 bp and N50 was 1,129 bp. The unigene size distribution showed the following: 68.54% (201,274) of the unigenes were between 200 and 500 bp in length; 15.55% (45,669) were between 500 and 1,000 bp; 5.64% (969) were between 1,000 and 1,500 bp; 3.60% (10,582) were between 1,500 and 2,000 bp; and 6.66% (19,568) were more than 3,000 bp in length.

**Table 1:** Overview of the datasets produced by RNA-seq(^a^ leaves, ^b^ stems, ^c^ roots)

	L^a^-1	-2	L-3	S ^b^-1	-2	S-3	R ^c^-1	R-2	R-3	all
Total raw reads	83,579,036	764,139,06	122,713,724	81,388,018	84,071,502	98,795,482	99,973,480	75,819,952	78,501,126	801,256,226
Total clean reads	75,806,512	68,952,464	109,571,128	73,441,140	76,130,792	89,106,624	90,308,382	68,347,296	70,709,828	722,374,166
Q20% (%)	99.24	99.20	99.27	99.23	99.24	99.26	99.20	99.24	99.20	
GC percentage (%)	46.05	46.29	46.46	97.86	97.88	97.93	46.03	45.71	46.76	

### Transcriptional profiling of *S. cusia* organs

Different expressed genes (DEGs) profiling between organs was carried out using Bioconductor. DEGs with significant transcriptional variation were screened with a threshold of FDR ≤ 0.05. Totally 729 DEGs were found between roots and stems, 3,489 between roots and leaves, and 3,003 between stems and leaves (Table S2). Moreover, good homogeneity of replicate samples for each organ was verified by hierarchical clustering of samples.

**Fig. 2:**
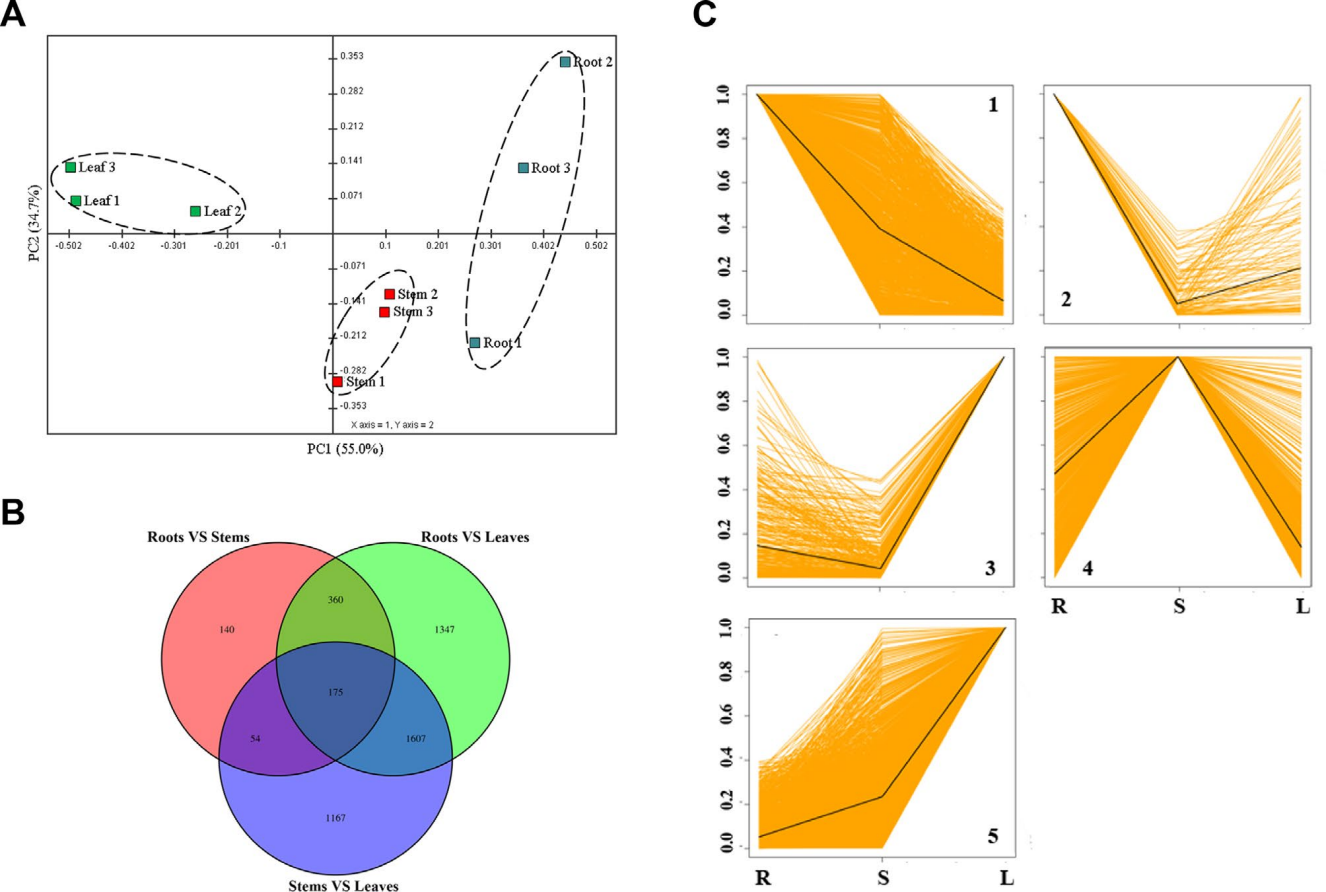
Comparison of differentially expressed genes in different *S. cusia* organs. **(A)** PCA represented transcriptional variation of all test samples. **(B)** Venn digrams showed numbers of common and specific genes to each organ. **(C)** All differentially expressed genes in *S. cusia* organs fall into five different major clusters based on similar patterns of expression (K-medoids clustering)

Principal component analysis (PCA) was performed to characterize each sample based on transcriptional variation and to indicate the affiliations within the transcriptional diversity. As shown, PC1 and PC2 explain 89.729% of the total transcriptional variation (Fig. 2A). Notably, samples of different organs represented significant separation, indicating dramatic transcriptional differences between different organs. Venn diagram further represented the distribution of DEGs between different organs (Fig. 2B). Two groups of comparison (stems VS leaves and roots VS leaves) showed most (1,682) DGEs in common. The DEGs distribution indicated less transcriptional variation between the roots and stems of *S. cusia*. Leaves showed much more specific transcription profile compared to other organs. To have an insight of DEGs distribution, all DEGs were grouped in clusters according to their spatial profiles using series clustering. All DEGs were grouped into five major clusters according to their organs expression profiles (Fig. 2C). Cluster 1 showed 559 DEGs that had high expression level in roots and low level in leaves. Cluster 2 grouped 44 DEGs that showed high expression level in roots and low level in stems. Cluster 3 (163 DEGs) and cluster 5 (1,149 DEGs) showed genes highly expressed in leaves but with different expression level in roots and stems. 445 genes grouped in cluster 4 showed peak expression level in stems. To further reveal functional specificity of DEGs in different organs, GO analysis of DEGs grouped in each cluster was carried out. Table S2 showed GO assignment of DEGs grouped in each cluster. According to metabolic traits, DEGs showed significantly high expression level in leaves, which mainly grouped in clusters 3 and 5, were focused. Metabolism related GO-terms as catalytic activity and metabolic process were significantly enriched. To further study the leaf specific metabolism, isogenes fall into these terms were searched for KEGG pathway assignment. As a result, there were no genes in cluster 3 matched to any KEGG pathway assignment related to indole metabolism. In cluster 5, ten unigenes majorly mapped into two indole metabolism pathways, they were tryptophan metabolism (ko00380) and phenylalanine, tyrosine and tryptophan biosynthesis (ko00400) [23, 24]. Obviously, all significant DEGs involved in indole synthesis fell into cluster 5, which should be paid attention for further understanding of indole metabolism in *S. cusia.*

### Identification and expression analysis of indole biosynthesis candidate genes

**Fig. 3:**
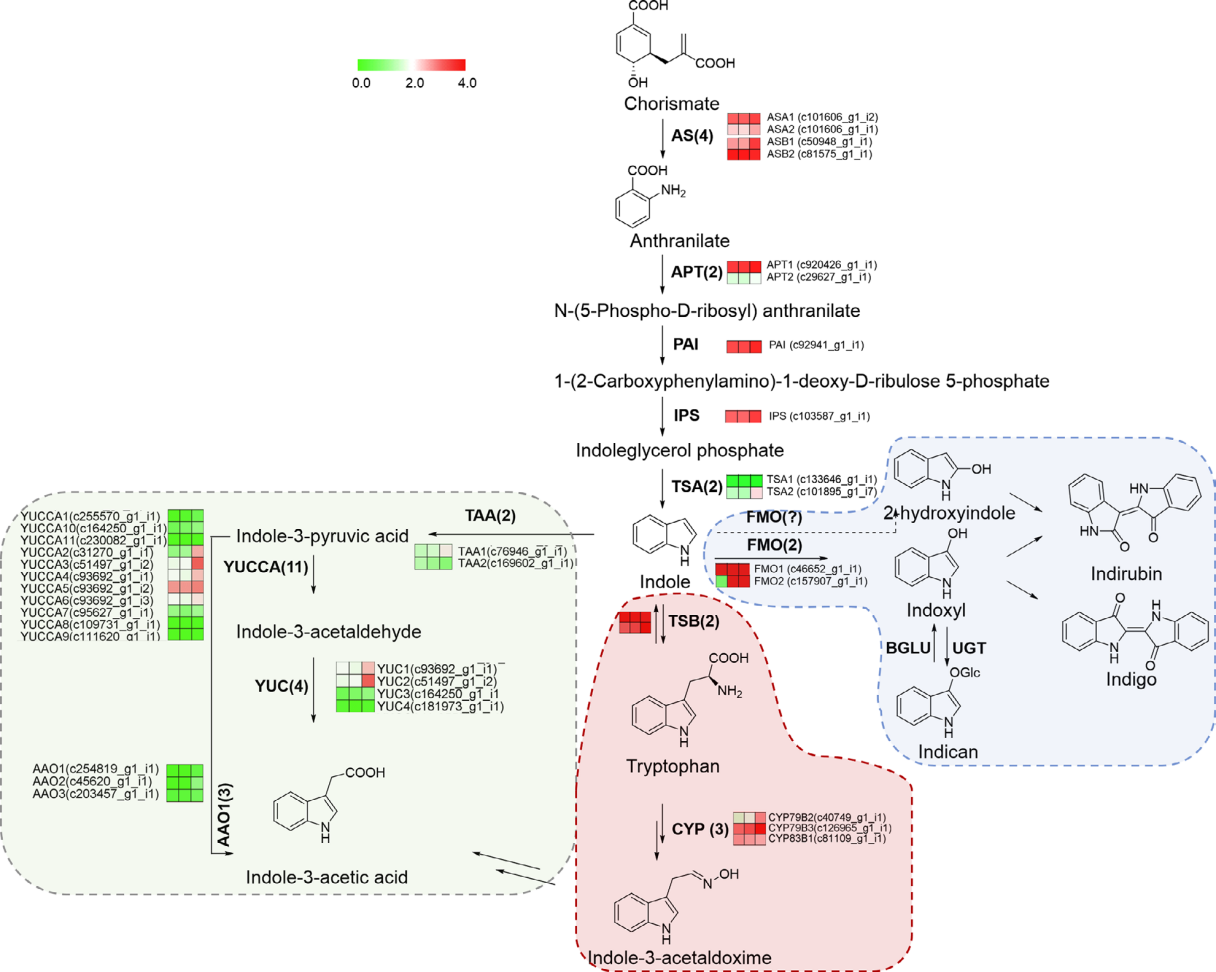
Putative indoles biosynthesis pathway in *S. cusia* and gene expression of enzymes involved. Different arrow color and background color indicated diverse metabolism branches, including indigo and indirubin (blue), indole-3-acetic acid (green), and indole-3-acetaldoxime(red). Heatmaps displaying the differential expression of transcripts encoding for enzymes involved in each catalytic step. Different columns represent tissues in order of roots, stems, and leaves. Color scale representing normalized expression values is shown. Anthranilate synthase, AS; Anthranilate phosphoribosyltransferase, APT; Phosphoribosylanthranilate isomerase, PAI; Indoleglycerol phosphate synthetase, IPS; Trpotophan synthase α subunit, TSA; cytochromeP450, CYP; Tryptophan aminotransferase, TAA; YUCCA (YUC) flavin-containing monooxygenase, YUC; Aldehyde oxidase, AAO; UDP-glucuronosyltransferases, UGT; flavin-dependent monooxygenase, FMO. We have been permitted to use the KEGG image of ko00380 and ko00400 from the rights holder

Biosynthesis of indigo in the plant is involved in indole biosynthesis pathways, which are part of complex tryptophan metabolism. Indole metabolism mainly constitutes by indole-3-acetaldoxime (IAOx) pathway, indole-3-acetic acid (IAA) pathway metabolism, and indigo synthesis. Using blast search against reference genes involved in indole metabolism, 37 unigenes coding 12 catalytic enzymes were predicted to be involved in the complex indole metabolism of *S. cusia* (Fig. 3). Multiple pathways contribute to IAA biosynthesis in plants [25]. According to transcriptome annotation, a totally 20 isogenes coding four catalytic enzymes that were involved in the indole-3-pyruvic acid (IPA) pathway and indole-3-acetaldoxime (IAOx) pathway was identified. Among them, indole-3-acetaldoxime (IAOx) was derived from tryptophan by three cytochrome P450 (CYPs) (CYP79B2/B3, 83B1). The IAOx pathway is not the main IAA biosynthetic pathway, it is mainly found in Brassicaceae, such as *Isatis indigotica, Arabidopsis thaliana* [26]. The synthetic mechanism of indigo in *S. cusia* is still unclear. Considering results from PtFMO [18] in *P. tinctorium*, we speculated that a flavin-dependent monooxygenase is also responsible for producing indoxyl from indole in *S. cusia*.

### Identification and biochemical characterisation of ScFMOs

**Fig. 4:**
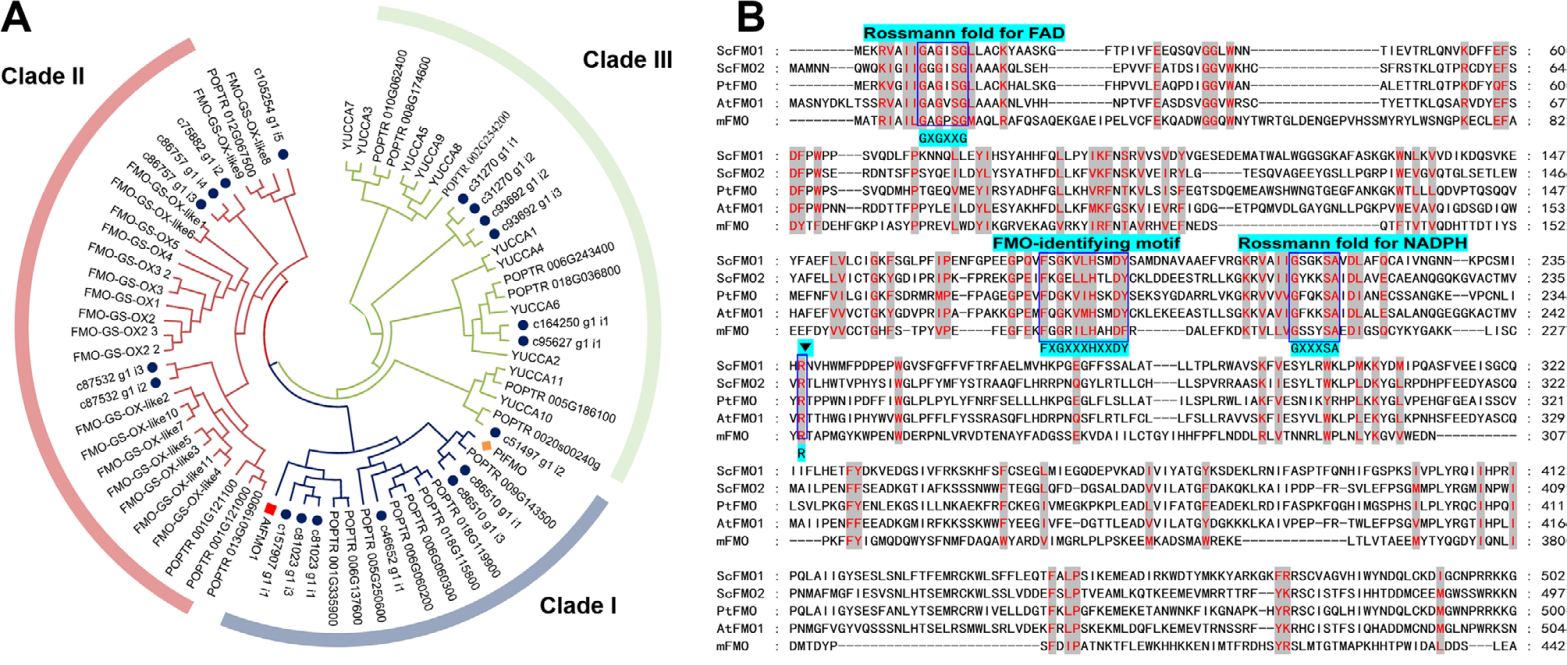
Identification and biochemical characterisation of ScFMOs. **(A)** Phylogenetic tree analysis of candidates ScFMOs and characterized model plant *Arabidopsis thaliana* and *Populus trichocarpa*. Bootstrap values (based on 1000 replicates) > 50% are indicated for their corresponding edges. ScFMO candidates are indicated as blue circle spot, orange diamond is PtFMO, red square is AtFMO1. **(B)** Mutiple sequence alignment of FMO from *S. cusia, P. tinctorium, A. thaliana and M. aminisulfidivorans.* The identical and similar residues in all of the proteins are shown as red words with gray background, respectively. The conserved residues of Rossmann fold for FAD and NADPH, FAD-identifying motif are highlighted as lake blue and dark blue box. The symbol inverted triangle display Arg-237 (R) residues in ScFMO1.

Using the transcriptome data mentioned above, we began to explore potential proteins that might be involved in indigo biosynthesis. Constructing local *S. cusia* protein database with NCBI BLAST software (ncbi-blast-2.9.0+-win64). HMM (Hidden Markov Model) profile of FMO conservative domain PF01494 was downloaded from (http://Pfam.xfam.org/), this file was used as a seed, Hmmer software is used to run HMMsearch in the local protein database, E-value was 0.01, moreover, 19 ScFMO of *S. cusia* were obtained. Phylogenetic trees of ScFMO and model plants *Arabidopsis thaliana* and *Populus trichocarpa* were constructed (Fig. 4A).

Several constitutive expressed showed high transcription level in leaves. Among them, two genes *FMO*1 and 2 (c46652_g1_i1, c157907_g1_i1) showed significant high transcription level in leaves and grouped into clade I by hierarchical clustering (Fig. 4A), which was further confirmed by qRT-PCR (Figure S6). The above organ specific expressed genes in leaves indicated their essential roles for the specific indole metabolism in *S. cusia* leaves. Two candidates share 52.8% and 38.0% amino acid sequence identity to the PtFMO. The full-length of two *ScFMOs* cDNA obtained 1575 bp and 1548 bp open reading frame and encoded 525 and 516 amino acid sequences, respectively. The corresponding ORF fragment was then amplified from *S. cusia* cDNA, and cloned into an *E. coli* expression vector for His_6_-tagged fusion protein overproduction. SDS-PAGE analysis of ScFMO1 and ScFMO2 expressions in *E. coli* revealed that the recombinant FMOs have a molecular mass of 60–70 kDa, which is close to the predicted molecular mass in Figure S7.

The amino acids sequence of ScFMOs contained commonly conserved motif in general FMOs: The FAD-binding domain (GXGXXG) at position 9–14 in ScFMO1, 14–19 in ScFMO2, and FMO-identifying motif (FXGXXXHXXDY) at position 179–189 in ScFMO1, 177–187 in ScFMO2. However, a Rossmann fold for NADPH showed some variation depending on FMO homologs. It was GxGxxG in FMOs from mammals, and yeast, whereas it was GxSxxA in FMOs from most bacteria [27]. By comparing two ScFMOs in *S. cusia*, PtFMO in *P. tinctorium* and AtFMO1 in *A. Thaliana*, we inferred the conserved motif of GXXKSA in plants (Fig. 4B).

LC-MS analysis results of enzyme assays indicated that indole was successfully converted into indoxyl, and then spontaneous oxidation to form indigo by ScFMO1 using FAD and NADPH as the cofactor (Figure S4). ScFMO2 shows no enzymatic activity.

### Indigo production in *E. coli*

**Fig. 5:**
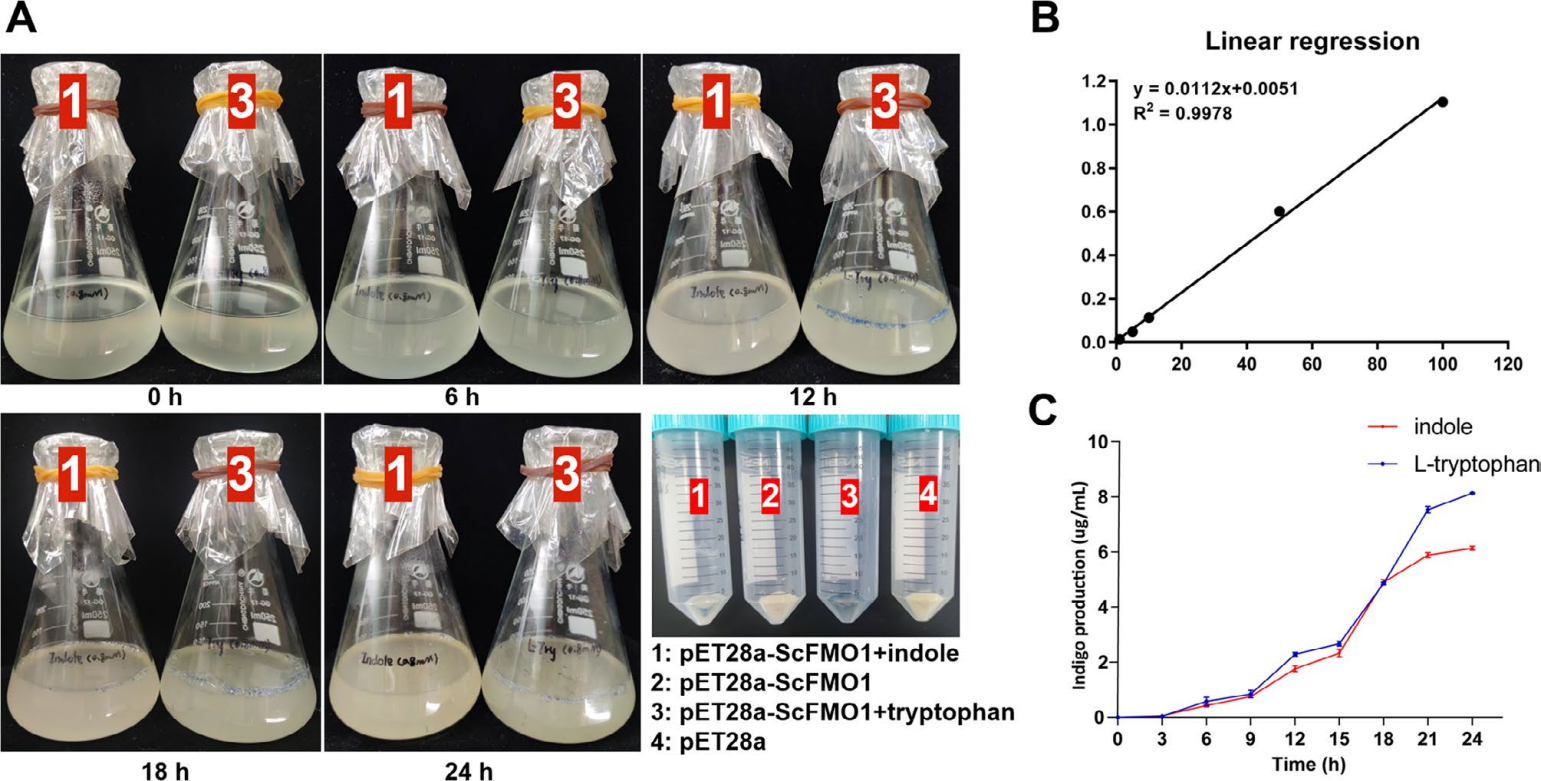
Expression of recombinant ScFMO and indigo production. **(A)** After induced by IPTG, the color of the culture was observed to change over time. The 2 and 4 shows *E. coli* harboring *pET28a* or *pET28a-ScFMO* without substrates that had been cultured for 24 h. **(B)** Linear regression curves of indigo by microplate absorbance reader with 630 nm filters. **(C)** Indigo production from indole and tryptophan in the culture over time

We constructed *pET28a-ScFMO1* and *pET28a-ScFMO2*, but only *pET28a-ScFMO1* could expressed with the addition of 0.1 mM IPTG to the medium. To examine whether ScFMO1 catalyzes the formation of indoxyl, we added tryptophan (0.8 mM) and indole (0.8 mM) to the expression medium, respectively, as substrates that may produce indoxyl directly or indirectly. As cultivation time increased, at 6 h after the addition of IPTG, the expression medium piecemeal produced blue stuff, in both substrates. After 24 h, the concentration of indigo in the expression medium was obtained about 8.12 µg·mL^− 1^ for tryptophan, and 6.63 µg·mL^− 1^ for indole (Fig. 5). The results demonstrated that the indigo color in centrifuge tube 3 (which used tryptophan as the substrate) was darker. No color change was observed in *E. coli* harboring empty pET28a vector and without substrates.

### Analysis of ScFMO1 subcellular localization

**Fig. 6:**
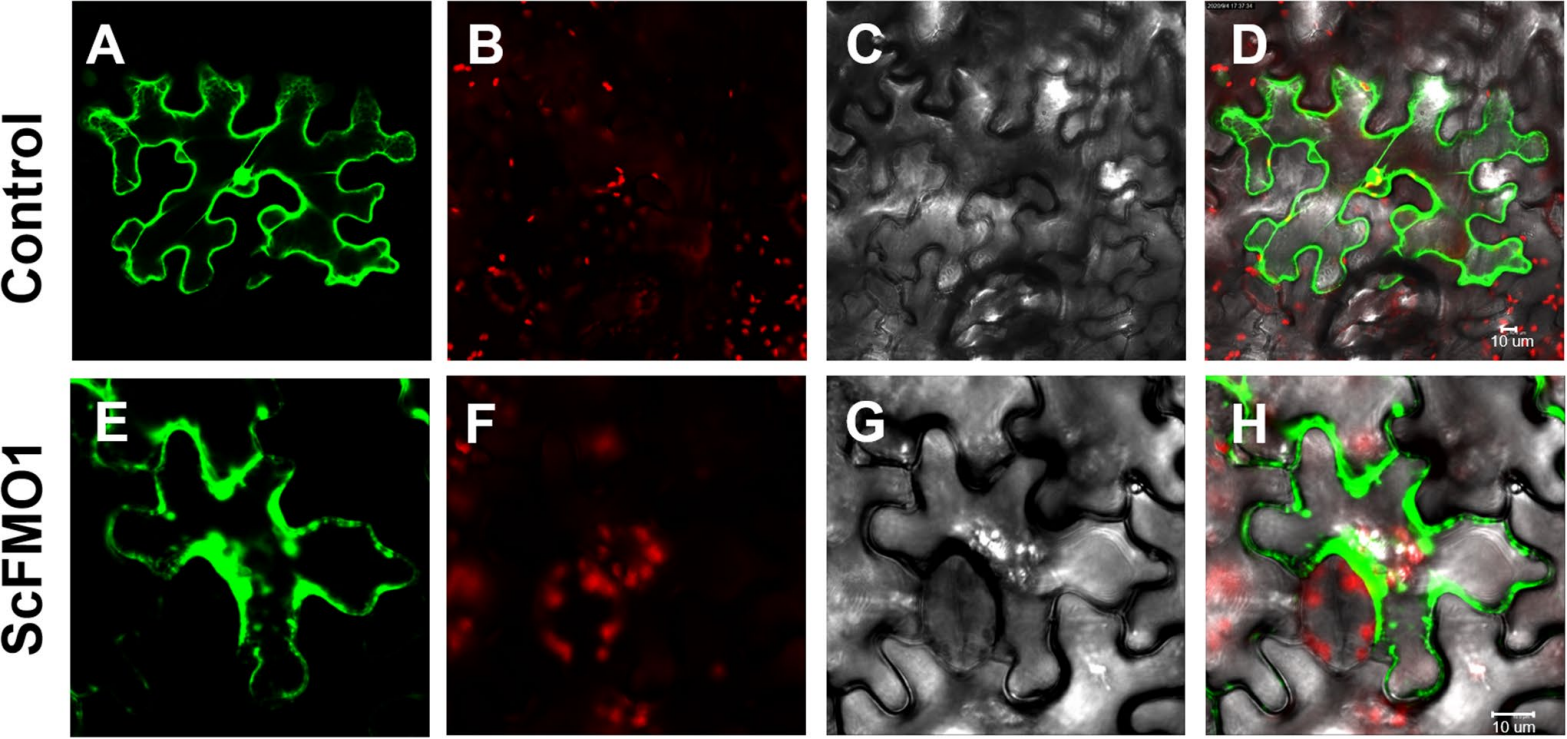
Subcellular localization of *ScFMO1-GFP*. **A-D**: Fluorescence micrographs of transgenic line expressing *pEAQ-eGFP* vector into tobacco leaves in under different fields. **E-H**: Fluorescence micrographs of transgenic line expressing *pEAQ-ScFMO1-eGFP* vector into tobacco leaves in under different fields. **(A, E)** show the green fluorescence of eGFP; **(B, F)** show the autofluorescence of chlorophyll; **(C, G)** show the bright filed; **(D, H)** are the merged image of (A, B and C), (E, F and G) ,

It is necessary to specify the site of indigo production. We pinpointed the ScFMO1’s subcellular location and clarified its biological roles using subcellular localization techniques. It is predicted that ScFMO1 may be located in the cytoplasm of the cell by the localization site (https://wolfpsort.hgc.jp/), the result was shown in Figure S5. To confirm this prediction, the *pEAQ-ScFMO1-GFP* vector was constructed and its validity verified by a transient expression experiment in tobacco leaves. It has been established that ScFMO1 localizes in cytoplasm but not in the cell wall or vacuole, as shown in Fig. 6E-H. The leaf cells of *S. cusia* generally have large vacuoles, the presence of vacuoles tends to squeeze the cytoplasm to the position close to the cell membrane, and at this time, we are unable to visualize the GFP signal covering the whole cell [28]. Figure 6 A-D shown that the signal *pEAQ-eGFP* alone were transformed into tobacco leaf and fluorescence was observed in the cell wall and nucleus. We may be more certain that ScFMO1 should perform its catalytic role in the cytoplasm since the production of indoxyl from indoles requires the involvement of O_2_.

## Discussion

### Indole biosynthesis pathway in *S.cusia*

Chorismate is the source of tryptophan, which is produced as the central branch point of the complete tryptophan metabolism. To have an overview of this intricate metabolism network, a probable biosynthetic pathway for indole metabolism was mapped in accordance with the transcriptome annotation. Three major branches were identified, including Indole-3-acetic acid (IAA), indole-3-acetaldoxime (IAOx), and indigo/indirubin synthesis (Fig. 3). In plants, IAA and indole-3-acetaldoxime synthesis are frequent and well-researched. From *S. cusia* transcriptome, the majority of the genes involved in the IAA and IAOx pathway were completely identified and presented as multi-gene families.

For insights into the yields of indigo and indirubin in *S. cusia*, we primarily focused on biosynthesis of indigo precursors in this work. According to the putative biosynthesis network in *S. cusia* (Fig. 3), TSA (using indole-3-glycerol phosphate as substrate) and TSB (using tryptophan as substrate) catalyze the initial steps forming indigo and indirubin. Two unigenes (c126492_g1_i1 and c90735_g1_i3) coding *TSA* genes and two TSB genes (c218344_g1_i1 and c94493_g1_i1) were discovered by our group.

Indoxyl, produced by the hydroxylation production of indole and catalyzed by monooxygenases, is the considered precursors to indigo and indirubin. Heterologous oxygenases can transform indole into 3-hydroxyindole (indoxyl), 2-hydroxyindole, isatin, and 2-oxindole [29, 30]. Two molecules of indoxyl spontaneously dimerize into indigo in the presence of oxygen, whereas indoxyl and 2-hydroxyindole are condensed into indirubin (in Fig. 3). Secondary metabolites are hydroxylated by CYPs and FMOs associated with the endoplasmic reticulum membrane. No related plant CYP has been reported as of yet. The human cytochrome P450 mono-oxygenase 2A6 (CYP2A6) was utilized as a reference since it may produce indigo in bacterial cultures [31]. A putative homologous gene of *CYP2A6* (c255055_g1_i1) from *S. cusia* transcriptome was predicted to catalyze this unique hydroxylation reaction. Unfortunately, we have not been able to characterize it. Therefore, we focused on the genes from the FMO family. Transcriptional profiles of all putative catalytic genes were represented in Fig. 3. Isoforms of each catalytic gene showed diverse expression patterns.

### Key enzyme genes of indigo biosynthesis

Only a few plant species, including *S. cusia* (Acanthaceae), *P. tinctorium* (Polygonaceae), *I. indigotica* and *I. tinctoria* (Brassicaceae), *Indigofera suffrutticosa* and *I. tinctoria* (Fabaceae), etc., have the ability to synthesize indigo and indirubin. Indigo and indirubin are typically thought to be impossible to find in living plants. They are generated following the fermentation of indigoferous plants and are not a natural component of the plants. The blue color eventually emerges as the leaves are damaged and the vacuoles (which contain indican) and chloroplasts (which contain β-glucosidase) are cleaved. The important precursors of indigo and indirubin are 3-hydroxyindole (indoxyl) and indican. In spite of the fact that indigo and indirubin were found in fresh aerial parts of *S. cusia*, they cannot be regarded as byproducts of catalytic reactions. They might be the products of indigo precursors after air oxidation. We suggested that the level of indigo and indirubin in *S. cusia* depend on the level of indigo precursors as indoxyl and indican.

The production of indoxyl and indican in plants is still poorly understood. The functional characterization of several enzymes from indigoferous plants has been reported, for example, PtFMO and PtUGT (PtIGS) from *P. tinctorium*, and ItUGT1/2 from *I. tinctoria* (Fabaceae). Although *S. cusia’*s genome and transcriptome have been described, genes associated with indigo biosynthesis were not yet characterized. Based on the foregoing, we have assumed the involvement of a monooxygenase that catalyzes the formation of indoxyl from indole. Hence, we clone the *ScFMO1* gene based on sequence data derived from RNA-Seq analysis.

The constructed phylogenetic trees of ScFMOs, PtFMO and model plant *A. thaliana* and *P. trichocarpa* fall into three cluster (Fig. 4A). ScFMO1, PtFMO, and AtFMO1 appears to be close relative to Clad I, the levels of sequence identity show about 52.8%, 37.0%, 16.5% amino acid sequence identity to the PtFMO, AtFMO1 and mFMO, respectively. ScFMO1 contains two Rossmann folds for FAD and NADPH, and the FMO-identifying motif FXGXXXHXXXY (Fig. 4B). In bacteria, two pivotal resides, Tyr-207 (Y) and Arg-229 (R), regarding the indole oxygenation [27]. In the plant, 214-Lys (K) for the NADPH domain (GXXKSA) show more consistent performance, Arg-237 (R) in ScFMO1 also forms part of entrance to the NADPH binding cavity, it seems to reflect differences between species.

The fermentation experiments of ScFMO1 had been conducted, and indigo could be produced when tryptophan and indole were used as substrates. However, we still believe that indole is the real substrate of ScFMO1, which has been confirmed by enzyme catalytic experiments. Tryptophan entered the metabolic pathway of *E. coli* during fermentation to produce indole, which was then used as the substrate of ScFMO1 to produce indoxyl.

## Conclusion

In this study, we provided insights into molecular mechanisms driving the metabolism of indole, relied on transcriptome and metabonomic analysis, we found a novel flavin-dependent monooxygenase in *S. cusia* (ScFMO1) that is proved to be able to create indoxyl from indole. There has been discussion of the parallels and differences between microbial and plant FMOs. These findings will help to elucidate the molecular basis of indole alkaloid biosynthesis, make structural modifications and develop strategies to increase their yield.

## Methods

### Plant materials

*S. cusia* was gathered from a farmland of in Xianyou County, Fujian Province, China. Deposited in our university seed bank and are freely accessible for research. Different organs, including roots, stems and leaves were collected separately from the plants and frozen immediately in liquid nitrogen and stored at − 80 °C.

### *De novo* assembly and Functional annotation

Total RNA from the three organs was isolated with TRIzol reagent (Tiangen, Beijing, China) and biological replicates three times. All mRNA samples were purified using oligo (dT) attachment beads and broken into small fragments in length of 100–400 bp. The cDNA library was constructed using a TruseqTM RNA sample prep Kit (Illumina, San Diego, CA). Sequencing was performed on an Illumina HiSeq2000 platform (Illumina). The raw RNA-seq read data are deposited in the Short Read Archive (http://www.ncbi.nlm.nih.gov/sra/). Short reads were assembly by trinity (http://trinityrnaseq.sourceforge.net/). Contigs were clustering by CD-HIT to produce longer contigs (unigenes).

All assembled unigenes were further analyzed to predict their relevant information. First, the unigenes were analyzed to predict assumed gene descriptions by searched in the protein databases (NCBI NR, Swiss-Prot, and clusters of orthologous groups (COG)). And then, based on the annotation, we make use of Blast2GO software to predict GO terms of molecular function, cellular component, and biological process. All assembled sequences were compared to the COG database to forecast and classification of possible gene functions based on orthologues. The KEGG pathways annotation was implemented by the KEGG Automatic Annotation Server (KAAS) (http:/www.genome.jp/tools/kaas/) (Figure S1, S2).

### Differentially expressed genes analysis

Clean reads were aligned against the unigene using RSEM (v 1.2.4), and expression values were calculated using FPKM (Reads Per Kilobase of exon model per Million mapped reads). The differentially expressed genes were analyzed in Bioconductor packages DESeq (V1.14.0). Double-layer standards were used to filter significantly differentially expressed genes (FDR ≥ 0.05). Transcriptional variation and clustering were presented by heatmap and were performed by MEV (v 4.9.0) (Figure S3).

### Expression tendency and co-expression analysis

According to the FPKM variation trend of different genes, a group of unique model expression trends were identified. The expression model profiles are related to the actual or the expected number of genes assigned to each model profile. Fisher’s exact test and multiple comparison test both showed higher than expected probability of significant distribution.

Co-expression network was built to represent the relations among genes. Networks were built according to the normalized expression values of genes selected from genes in significant GO terms. For each pair of genes, the Pearson correlation was calculated in two ways ANOVA using SPSS (v IBM SPSS Statistics 21.0). The co-expression correlation coefficient matrix was performed by MEV (v 4.9.0). For the co-expression correlation network, gene pairs were chosen with a threshold ≥ 0.99. Visualization of correlation network was performed using NetDraw (v 2.084).

### Metabolic analysis

*S. cusia* organs were dried in a constant temperature drying oven at 60 °C, and powdered. Chemical extraction of each sample (10 mg for each sample) was extracted by ultrasound extraction for three times (30 min) using solvent consisting of *N*,*N*-Dimethylformamide: methanol (1:1, v/v) with a final volume of 10 mL. Chemical standards of indigo, indirubin and tryptanthrin were purchased at Sigma-Aldrich (St. Louis, MO). The chemical constituents were detected by liquid chromatography (Agilent 1290, Agilent Technologies, Santa Clara, CA). Gradient elution chromatography was applied. A mobile phase consisting of methanol: water with 0.2% formic acid solution (40-95%, v/v) was used, with the flow rate is set to 1 mL·min^− 1^ and the run time is 30 min. The detection wavelength was 289 nm. Detection and quantification of compounds compared with authentic standard curves and retention times (RT, Tryptanthrin, 9.53 min, Indigo, 12.850 min, and Indirubin, 16.640 min. Figure S8).

### Sequence alignment and phylogenetic analysis

The candidate sequences obtained from the transcriptome were analyzed, and FMO families in model plants were selected for evolutionary tree alignment analysis. Firstly, multi-sequence alignment of protein sequences was performed using Clustal X2 software, and then phylogeny was inferred using the maximum likelihood method of default parameters in software MEGA-X. GenBank accession numbers for sequences used in constructing the FMO phylogenetic tree: PtFMO, BCL56286.2; AtFMO1, NP_173359.3; POPTR_006G137600, PNT31564.1; POPTR_001G335900, PNT58054.1; POPTR_018G119900, PNS94014.1; POPTR_018G115800, PNS93967.1; POPTR_006G060300, PNT30015.1; POPTR_006G060200, PNT30014.1; POPTR_009G143500, PNT21359.1; POPTR_005G250600, PNT38642.1; POPTR_013G019900, PNT06235.1; POPTR_001G121000, PNT54093.1; POPTR_001G121100, PNT54095.1; FMO-GS-OX1, NP_176761.1; FMO-GS-OX3, OAP12395.1; FMO-GS-OX5, NP_001323223.1; FMO-GS-OX2, ANM58465.1; FMO-GS-OX4, OAP15384.1; FMO-GS-OX-like7, NP_001321123.1; FMO-GS-OX-like 6, NP_172677.1; FMO-GS-OX-like1, NP_172680.1; FMO-GS-OX-like8, NP_200937.1; FMO-GS-OX-like4, NP_176448.1; FMO-GS-OX-like10, Q9C8T8.3; FMO-GS-OX-like3, NP_176450.2; FMO-GS-OX-like5, NP_176526.1; FMO-GS-OX-like2, NP_172684.1; FMO-GS-OX-like11, NP_001319303.1; FMO-GS-OX2(2), NP_001320895.1; FMO-GS-OX3(2), NP_001321026.1; FMO-GS-OX3(3), NP_001320894.1; FMO-GS-OX-like9, KAG7608478.1; POPTR_012G067500, PNT09842.1; YUCCA1, NP_194980.1; YUCCA4, KAG7601911.1; YUCCA8, NP_194601.1; YUCCA6, NP_197944.2; YUCCA2, KAG7615797.1; YUCCA7, NP_180881.1; YUCCA5, NP_199202.1; YUCCA11, NP_173564.1; YUCCA10, NP_175321.1; YUCCA9, NP_171914.1; YUCCA3, NP_171955.1; POPTR_006G243400, PNT33509.1; POPTR_018G036800, PNS92511.1; POPTR_002G254200, PNT51657.1; POPTR_010G062400, RQO96322.1; POPTR_008G174600, PNT25234.2; POPTR_005G186100, PNT37399.1; POPTR_0020s00240g, XP_006389665.1.

### qRT-PCR validation

To check the expression level of several candidate genes, tissue samples including leaf, stem and root were collected, and three independent replicates of each tissue were used. Total RNA was isolated from each sample using the MagicPure® Total RNA Kit (TransGen Biotech Co., LTD., Beijing, China). The cDNA was synthesized and then was used for the qRT-PCR reaction using PerfectStart® Uni RT&qPCR Kit (TransGen Biotech Co., LTD., Beijing, China). The *ACTIN2* gene served as an internal reference [32].

### Molecular cloning and overexpression of recombinant proteins

Gene-specific primers are used to amplify the coding sequences of the candidate genes from cDNA by PCR (Table S1). The amplicons were inserted into the Hind III site and EcoR I site of pET28a vector by ClonExpress II One Step Cloning Kit (Vazyme Biotech Co., Nanjing, China). *E. coli* BL21(DE3) transform with appropriate constructs were grown in Lysogeny Broth (LB) 5 mL containing 50 µg·mL^− 1^ kanamycin at 37 °C, 220 rpm, until the OD_600_ reached 0.6. And then transfer to 100 mL M9 medium (containing Na_2_HPO_4_.12H_2_O 17.08 g·L^− 1^, KH_2_PO_4_ 3.0 g·L^− 1^, NaCl 0.5 g·L^− 1^, NH_4_Cl 1.0 g·L^− 1^, MgSO_4_ 2 mM, CaCl_2_ 0.1 mM, nicotinic acid 1.0 µM, riboflavin 1.0 µM, 0.2% glycerol) [18]. After induced by 0.1mM Isopropyl-β-D-thiogalactoside (IPTG), and then let the culture continue to grow for 20 h at 25 °C, 220 rpm. The cells were collected by centrifugation and resuspended in a lysis buffer (20 mM Tris–HCl, pH = 7.8, 200 mM NaCl), and an Ultrasonic Cell Disruptor was used to crack the *E. coli.* The cracking conditions were as follows: power 30%, ultrasonic 3 s, gap 2 s, 40 Hz, 3 min, kept in ice water. The supernatant of the crude protein lysates was centrifuged, then filtered, and finally, gravity chromatography was performed with nickel–nitrilotriacetic acid (Ni–NTA). After loading the lysate, a 10-column volume (CV) of lysis buffer was first used, followed by 2-CV of elution buffer (50 mM Tris–HCl, pH 7.8, 150 mM NaCl and 25 mM-300 mM imidazole) to obtain His-tagged recombinant protein. Based on the results of SDS-PAGE, the required protein components were combined, desalted by dialysis using Amicon Ultra-15 Centrifugal Filters (Millipore), concentrated and stored in storage buffer (50 mM Tris–HCl, pH 7.8, 150 mM NaCl, and 50% glycerol) in -80 °C.

### Indigo production in *E. coli* from tryptophan and indole

*pET28a-ScFMO* was transformed into *E. coli* BL21(DE3) was cultivated in the LB medium. After the expanded culture, tryptophan and indole were respectively added to the expression M9 medium, with a final concentration of 0.8 mM. And then induced with 0.1 mM IPTG when the concentration of expression medium reaches OD 0.6. The cultures were incubated for 24 h at 25 °C, 2 mL of the bacteria will be absorbed from the culture medium at an interval of 3 h. Indigo was precipitated by centrifuging the culture medium at 10,000 × g for 10 min, washed at least 3 times with dd H_2_O, and dried under a vacuum for 6 h. Finally, the solution was dissolved in 1 mL of DMSO. The amount of bio-indigo was estimated by Bio-Rad iMark Microplate Absorbance Reader with 630 nm filters (Bio-Rad, California, USA). Commercial indigo dissolved in DMSO at 100, 50, 10, 5, 1 µg·mL^− 1^ were used as the standard curve, y = 0.0112x + 0.0051, R^2^ = 0.9987.

### Enzyme assay and LC-MS analysis

The assay mixture (200 µL) containing 1 mg crude protein extracts, 0.1 mM indole or tryptophan, 1 mM NADPH, 1 mM FAD were incubated at 30 °C with a shake gently at 60 rmp for 2 h. And then 200 µL ice methanol was used to terminate the reaction. The supernatant layer of the reaction mixtures is obtained by centrifugation at 10,000 g at room temperature for 10 min prior to analysis by LC-MS using an Agilent 1290 Infinity-6538 UHD and Accurate-Mass QTOF/MS (Agilent, Santa Clara, CA). Gradient elution chromatography was applied. A mobile phase methanol-water solution (10-90%, v/v) was used, with the flow rate set to 0.4 mL·min^− 1^ and the run time is 15 min.

### Subcellular localization of ScFMO1

As the object of transient expression experiment, we constructed the full-length *ScFMO1* cDNA obtained above into the vector containing GFP tag (*pEAQ-eGFP* plasmid). Then the *pEAQ-ScFMO1-GFP* constructs and the construct containing GFP alone were transformed into GV3101 Chemically Competent Cell, injected into tobacco for transient expression, and then the GFP and RFP fluorescence of cells are observed under fluorescence confocal microscope (Leica TCS SP5). GFP fluorescent signals were imaged at the excitation wavelength of 488 nm and emission wavelength of 505–530 nm. The red autofluorescence of chlorophylls was imaged at an emission wavelength longer than 680 nm.

### Electronic supplementary material

Below is the link to the electronic supplementary material.

Supplementary Material 1

## Data Availability

I can confirm I have included a statement regarding data and material availability in the declaration section of my manuscript. All RNA-seq reads generated by this study are publicly available at the NCBI Short Read Archive (SRA) under accession number SRR23424624 (https://dataview.ncbi.nlm.nih.gov/object/42163775) and BioProject accession PRJNA933948 (https://dataview.ncbi.nlm.nih.gov/object/PRJNA933948?reviewer=mkdvqsdek7547mj3g11trp9mmq).
