# Correction: Identification of novel flavin-dependent monooxygenase from Strobilanthes Cusia reveals molecular basis of indoles’ biosynthetic logic

**DOI:** 10.1186/s12870-023-04603-2

**Published:** 2023-11-22

**Authors:** Chang Liu, Mengya Cheng, Chao Ma, Junfeng Chen, Hexin Tan

**Affiliations:** 1 Department Chinese Medicine Authentication, College of Pharmacy, Naval Medical University (Second Military Medical University), Shanghai, China; 2 Department of Pharmacy, Shanghai Fourth People’s Hospital Affiliated to Tongji University School of Medicine, Shanghai, China; 3 School of Health Science and Engineering, University of Shanghai for Science and Technology, Shanghai, China; 4 Department of Vascular Disease, Shanghai TCM-Integrated Hospital, Shanghai University of Traditional Chinese Medicine, Shanghai, China; 5 Shanghai Key Laboratory for Pharmaceutical Metabolite Research, Shanghai, China

**Correction**: ***BMC Plant Biol*** **23, 527 (2023)**

**https://doi.org/10.1186/s12870-023-04557-5**

Following publication of the original article [1], author spotted errors in the affiliation of authors and their corresponding affiliation details. The correct affiliations are listed below:

Chang Liu1,2†, Mengya Cheng1,3†, Chao Ma4, Junfeng Chen1 and Hexin Tan1,2,5*

1Department Chinese Medicine Authentication, College of Pharmacy, Naval Medical University (Second Military Medical University), Shanghai, China

2Department of Pharmacy, Shanghai Fourth People’s Hospital Affiliated to Tongji University School of Medicine, Shanghai, China

3School of Health Science and Engineering, University of Shanghai for Science and Technology, Shanghai, China

4Department of Vascular Disease, Shanghai TCM-Integrated Hospital, Shanghai University of Traditional Chinese Medicine, Shanghai, China

5 Shanghai Key Laboratory for Pharmaceutical Metabolite Research, Shanghai, China

Furthermore, the font’s style and size of texts in the images of all figures are updated to be consistent. The correct Figs. 1, 2, 3, 4, 5, 6 are given below:

**Fig. 1:**
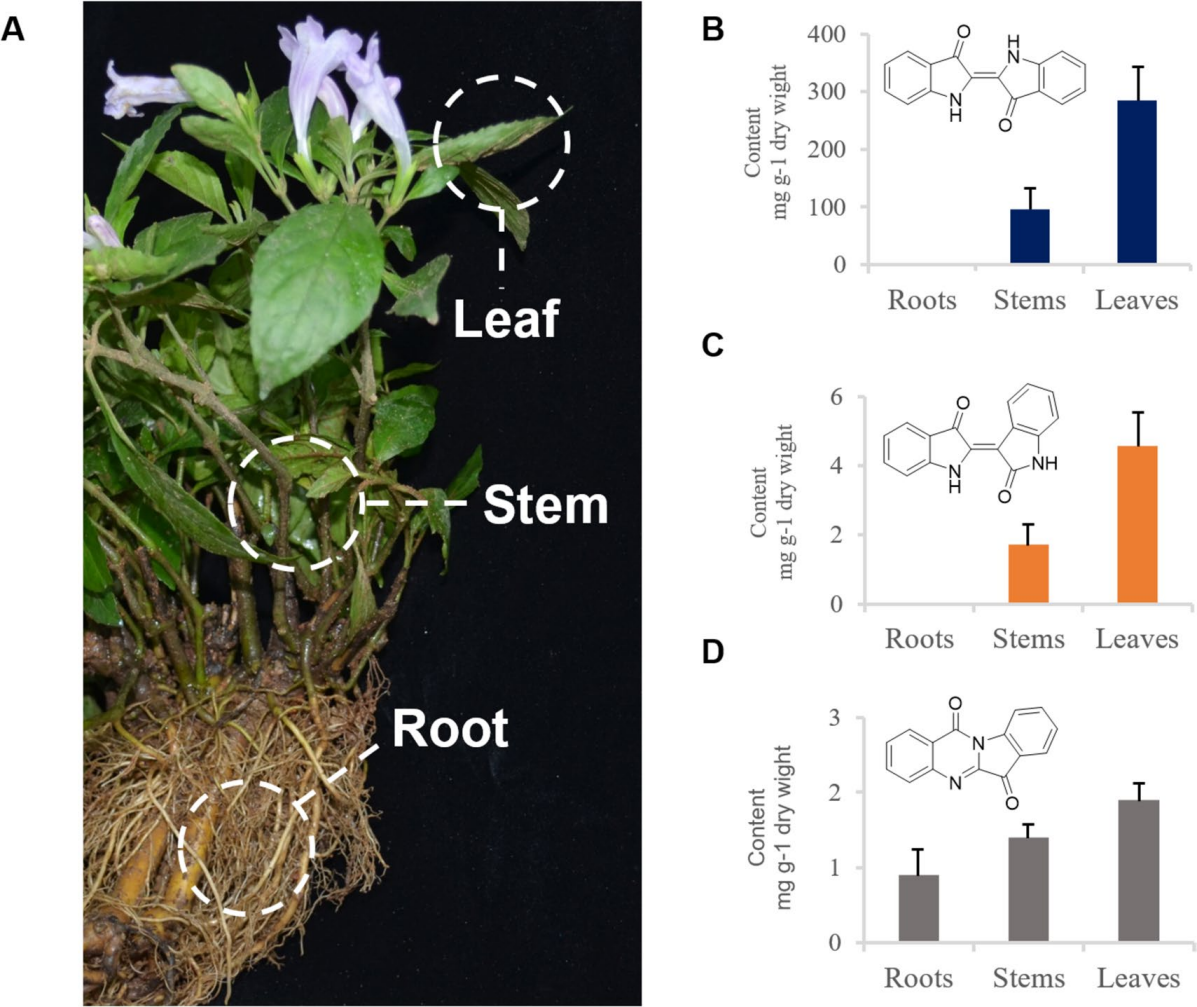
Accumulation of indigo and indirubin in different *S. cusia* organs. Mature plant of *S. cusia* (**A**). Accumulation patterns of indigo (**B**), indirubin (**C**), and tryptanthrin (**D**) in different *S. cusia* organs. Bars in blue indicated content level of indigo, and orange bars indicated that of indirubin. ND, not detected

**Fig. 2:**
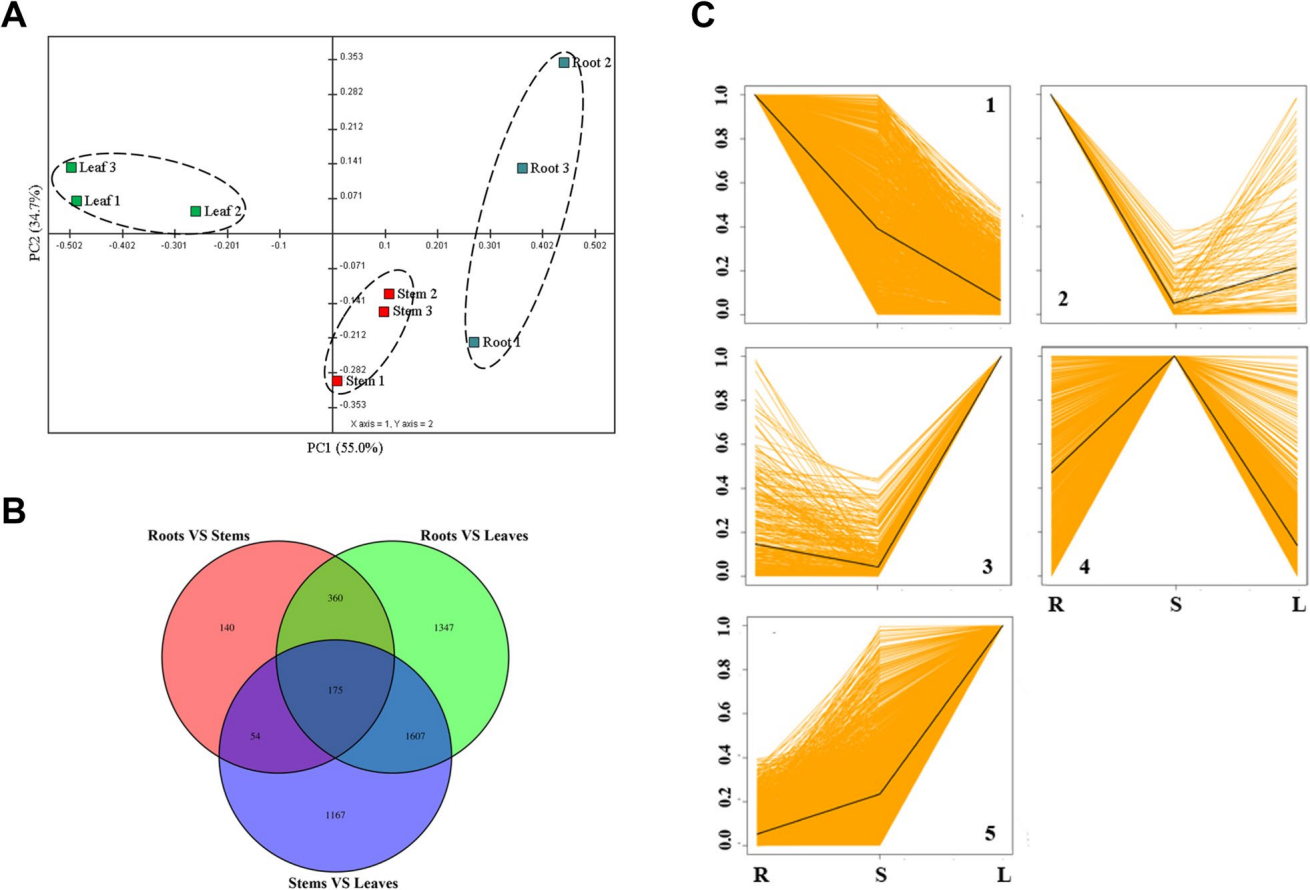
Comparison of differentially expressed genes in different *S. cusia* organs. (**A**) PCA represented transcriptional variation of all test samples. (**B**) Venn digrams showed numbers of common and specific genes to each organ. (**C**) All differentially expressed genes in *S. cusia* organs fall into five different major clusters based on similar patterns of expression (K-medoids clustering)

**Fig. 3:**
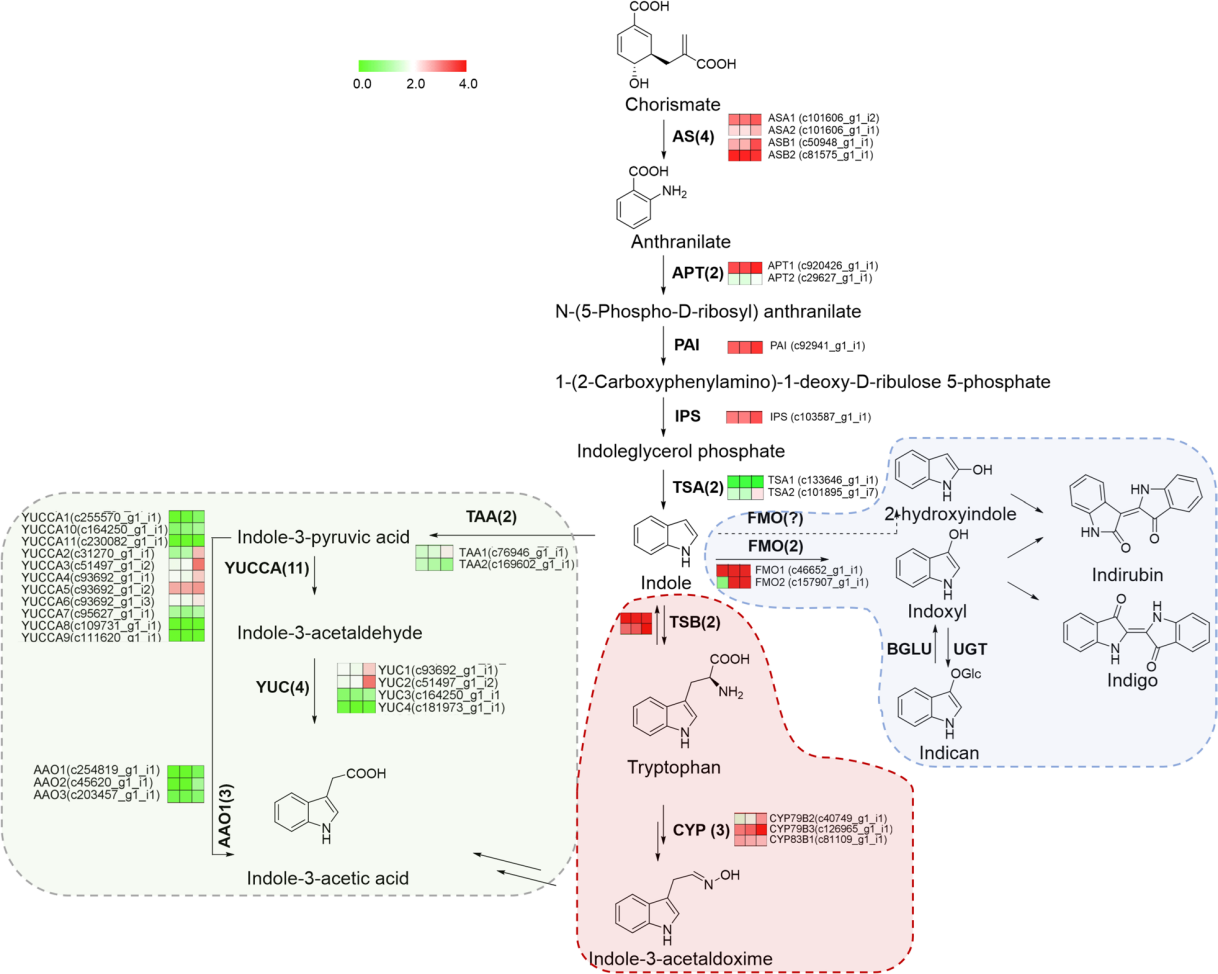
Putative indoles biosynthesis pathway in *S. cusia* and gene expression of enzymes involved. Different arrow color and background color indicated diverse metabolism branches, including indigo and indirubin (blue), indole-3-acetic acid (green), and indole-3-acetaldoxime (red). Heatmaps displaying the differential expression of transcripts encoding for enzymes involved in each catalytic step. Different columns represent tissues in order of roots, stems, and leaves. Color scale representing normalized expression values is shown. Anthranilate synthase, AS; Anthranilate phosphoribosyltransferase, APT; Phosphoribosylanthranilate isomerase, PAI; Indoleglycerol phosphate synthetase, IPS; Trpotophan synthase α subunit, TSA; cytochromeP450, CYP; Tryptophan aminotransferase, TAA; YUCCA (YUC) flavin-containing monooxygenase, YUC; Aldehyde oxidase, AAO; UDP-glucuronosyltransferases, UGT; flavin-dependent monooxygenase, FMO. We have been permitted to use the KEGG image of ko00380 and ko00400 from the rights holder

**Fig. 4:**
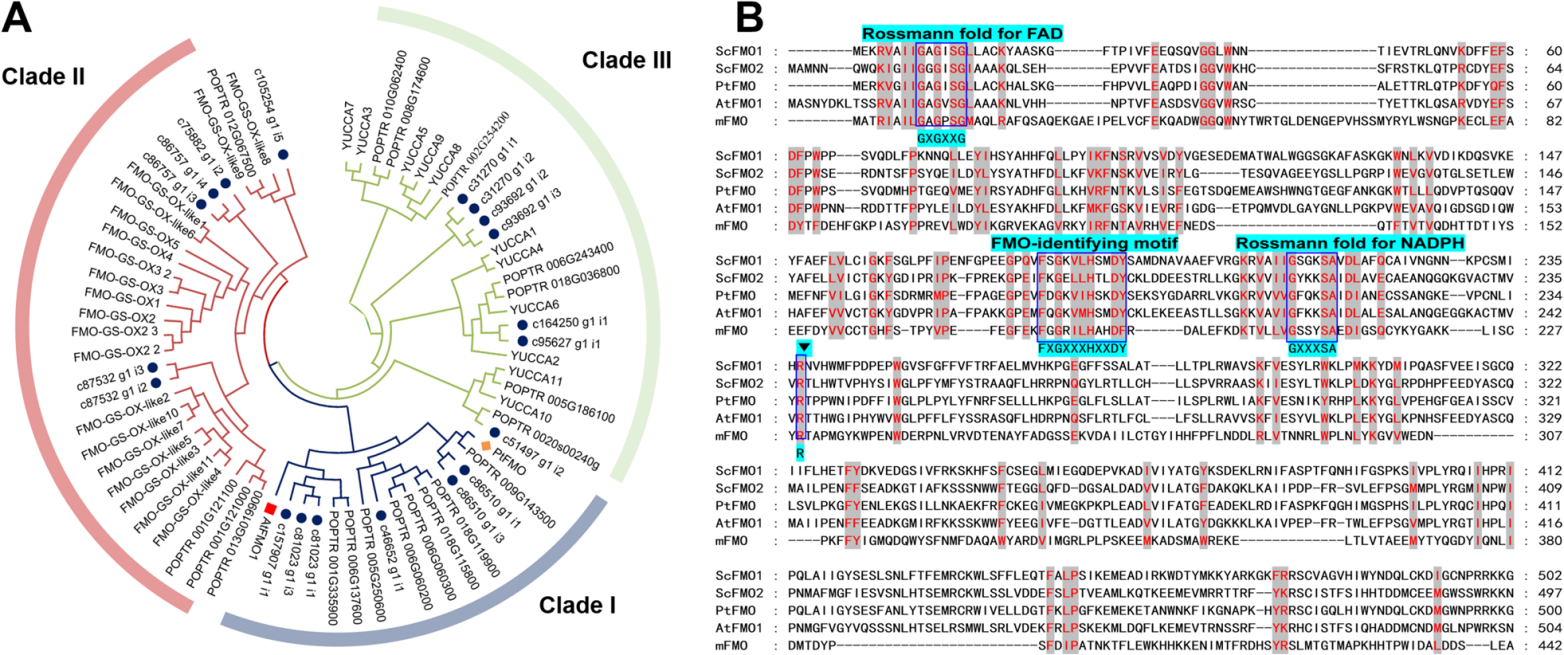
Identification and biochemical characterisation of ScFMOs. (**A**) Phylogenetic tree analysis of candidates ScFMOs and characterized model plant *Arabidopsis thaliana* and *Populus trichocarpa*. Bootstrap values (based on 1000 replicates) > 50% are indicated for their corresponding edges. ScFMO candidates are indicated as blue circle spot, orange diamond is PtFMO, red square is AtFMO1. (**B**) Mutiple sequence alignment of FMO from *S. cusia*, *P. tinctorium*, *A. thaliana* and *M. aminisulfidivorans*. The identical and similar residues in all of the proteins are shown as red words with gray background, respectively. The conserved residues of Rossmann fold for FAD and NADPH, FAD-identifying motif are highlighted as lake blue and dark blue box. The symbol inverted triangle display Arg-237 (R) residues in ScFMO1

**Fig. 5:**
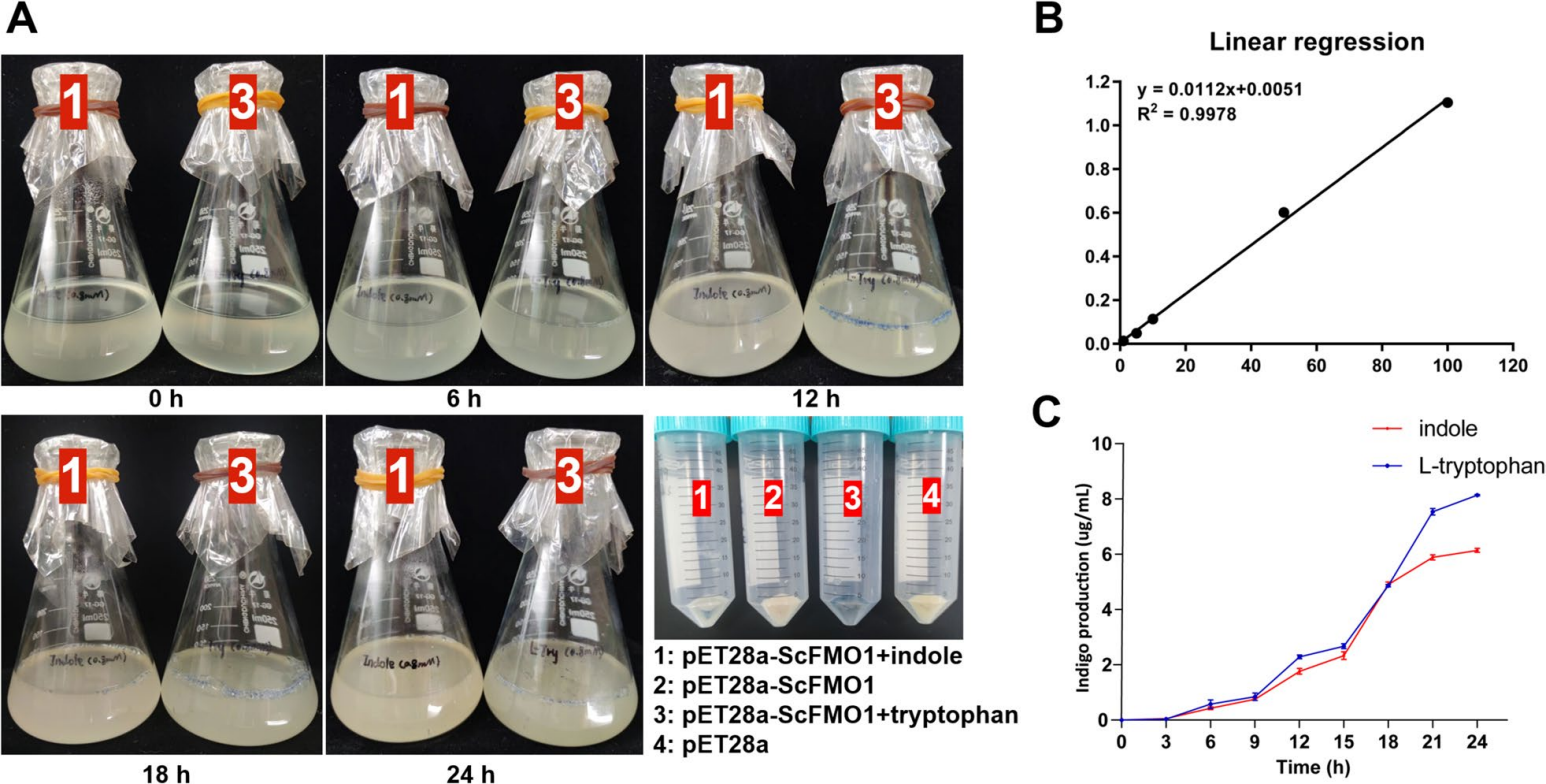
Expression of recombinant ScFMO and indigo production. (**A**) After induced by IPTG, the color of the culture was observed to change over time. The 2 and 4 shows *E. coli* harboring *pET28a* or *pET28a-ScFMO* without substrates that had been cultured for 24 h. (**B**) Linear regression curves of indigo by microplate absorbance reader with 630 nm filters. (**C**) Indigo production from indole and tryptophan in the culture over time

**Fig. 6:**
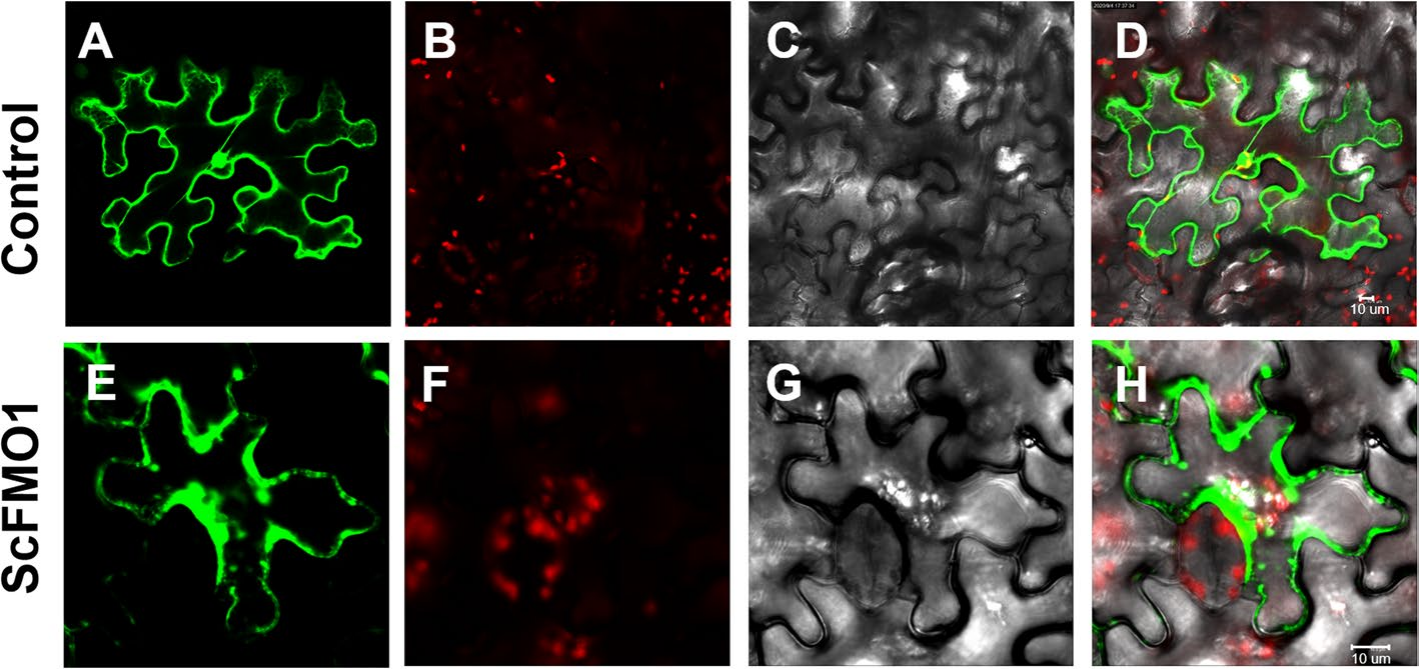
Subcellular localization of *ScFMO1-GFP*. **A-D**: Fluorescence micrographs of transgenic line expressing *pEAQ-eGFP* vector into tobacco leaves in under different fields. **E–H**: Fluorescence micrographs of transgenic line expressing *pEAQ-ScFMO1-eGFP* vector into tobacco leaves in under different fields. (**A, E**) show the green fluorescence of eGFP; (**B, F**) show the autofluorescence of chlorophyll; (**C, G**) show the bright filed; (**D, H**) are the merged image of (A, B and C), (E, F and G)

## References

[1] Liu C, Cheng M, Ma C (2023). Identification of novel flavin-dependent monooxygenase from *Strobilanthes Cusia* reveals molecular basis of indoles’ biosynthetic logic. BMC Plant Biol.

