# Supplementary material for: Identification of novel flavin-dependent monooxygenase from Strobilanthes Cusia reveals molecular basis of indoles’ biosynthetic logic
Source: BMC Plant Biol. 2023 Oct 31;23:527. doi: 10.1186/s12870-023-04557-5 (PMC10617207; doi:10.1186/s12870-023-04557-5)
Supplement: Supplementary file 1 — Supplementary Material 1 [file 12870_2023_4557_MOESM1_ESM.docx]

**Supporting Information**

**Identification of Novel Flavin-dependent Monooxygenase from Strobilanthes cusia Reveals Molecular Basis of Indoles’ Biosynthetic Logic**

Chang Liu^1,^ ^2, 5†^, Mengya Cheng^1,^ ^3†^, Chao Ma^4^, Junfeng Chen^1^, Hexin Tan^1,^ ^2, 5^*.

^1^Department Chinese Medicine Authentication, College of Pharmacy, Naval Medical University (Second Military Medical University), Shanghai, China

^2^Department of Pharmacy, Shanghai Fourth People’s Hospital Affiliated to Tongji University School of Medicine, Shanghai, China

^3^School of Health Science and Engineering，University of Shanghai for Science and Technology, Shanghai, China

^4^Department of Vascular Disease, Shanghai TCM-Integrated Hospital, Shanghai University of Traditional Chinese Medicine, Shanghai 200082, China.

^5^Shanghai Key Laboratory for Pharmaceutical Metabolite Research, Shanghai, China

^†^ These authors have contributed equally to this work.

^*^ Correspondence: Hexin Tan ([hexintan@163.com](mailto:hexintan@163.com))

**Table S1** The gene-specific primers for candidate genes were amplified from cDNA.

| Primers | 5'→3' |
| --- | --- |
| ScFMO1F | GGAGAAGAGGGTGGCAATAATC |
| ScFMO1R | CAGAGCTGATGAGGACTAATTCCG |
| ScFMO2F | GGCCATGAATAATCAGTGGCAG |
| ScFMO2R | TTTTTCTTCGTTGTAGTCTTGGCTG |

**Table S2** Number of DEGs in different organs.

|  | up | down | total |
| --- | --- | --- | --- |
| Root vs Stem | 366 | 363 | 729 |
| Root vs Leaf | 2056 | 1433 | 3489 |
| Stem vs Leaf | 1689 | 1314 | 3003 |


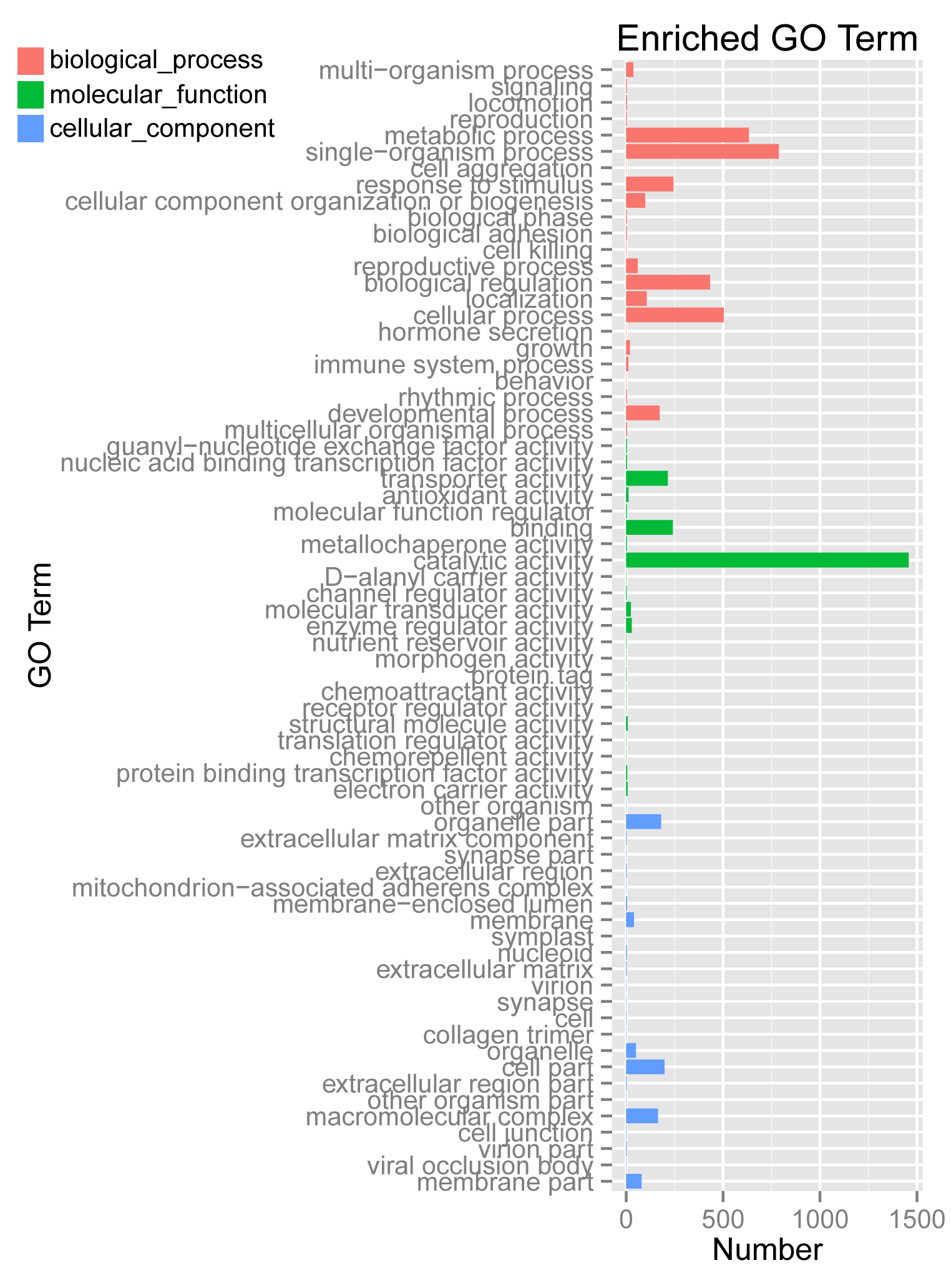


**Figure S1**. Gene Ontology (GO) category of all unigenes in *S. cusia* transcriptome.


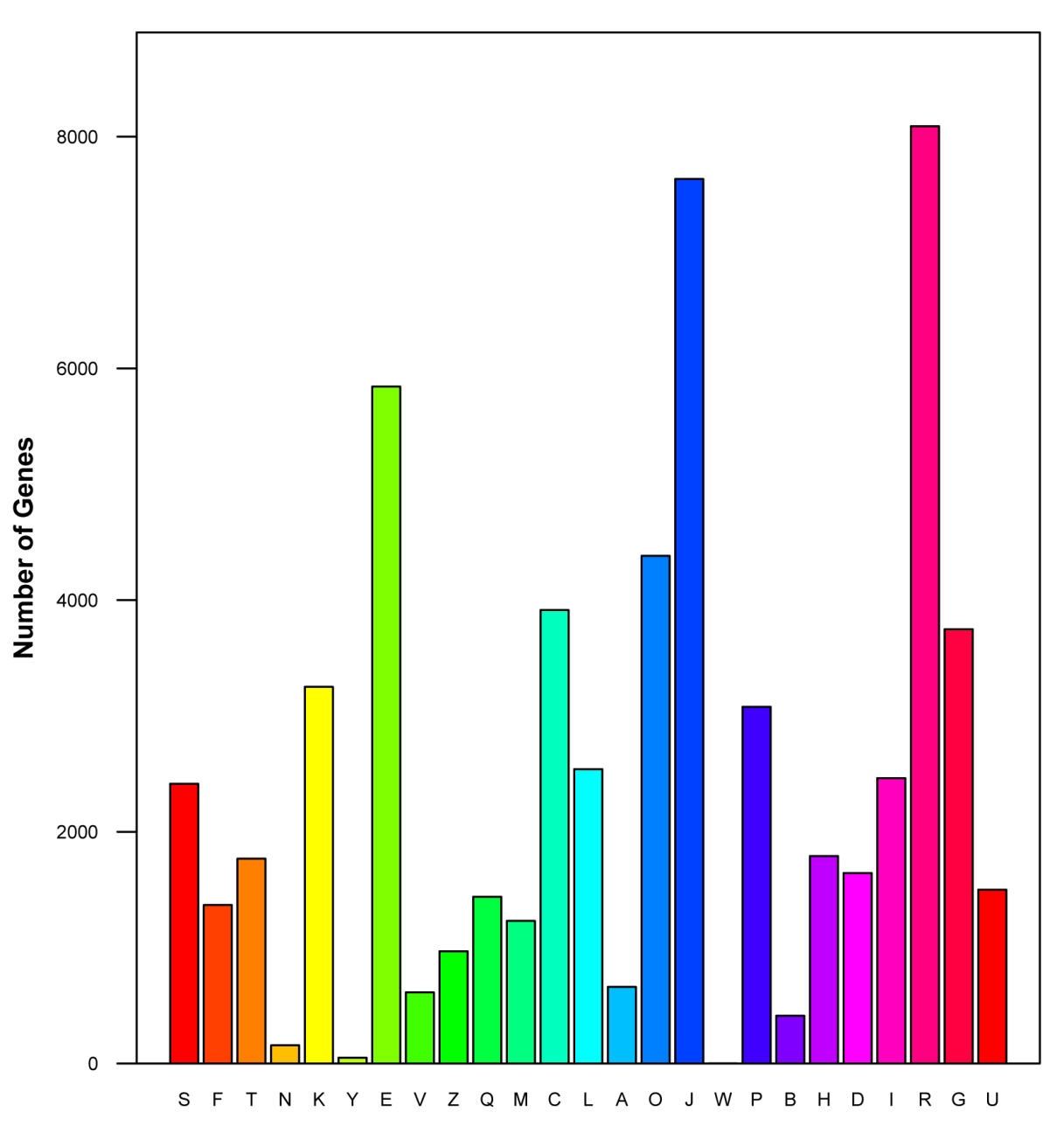


**Figure** **S2**. COG of all unigenes in *S. cusia* transcriptome.S, Function unknown. F, Nucleotide transport and metabolism. T, Signal transduction mechanisms. N, Cell motility. K, Transcription. Y, Nuclear structure. E, Amino acid transport and metabolism. V, Defense mechanisms. Z, Cytoskeleton. Q, Secondary metabolites biosynthesis, transport and catabolism. M, Cell wall/membrane/envelope biogenesis. C, Energy production and conversion. L, Replication, recombination and repair. A, RNA processing and modification. O, Posttranslational modification, protein turnover, chaperones. J, Translation, ribosomal structure and biogenesis. W, Extracellular structures. P, Inorganic ion transport and metabolism. B, Chromatin structure and dynamics. H, Coenzyme transport and metabolism. D, Cell cycle control, cell division, chromosome partitioning. I, Lipid transport and metabolism. R, General function prediction only. G, Carbohydrate transport and metabolism. U, Intracellular trafficking, secretion, and vesicular transport.


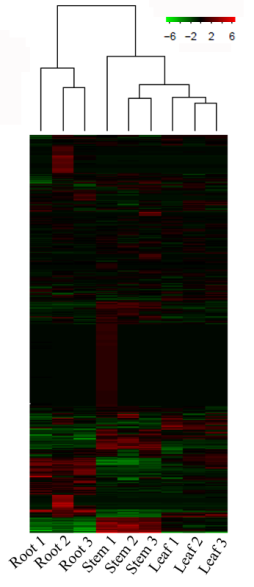


**Figure S3**. Heatmap of all different expressed genes (DEGs) in different organ samples represented more similar transcriptional variation of stems and leaves compared to that of roots.


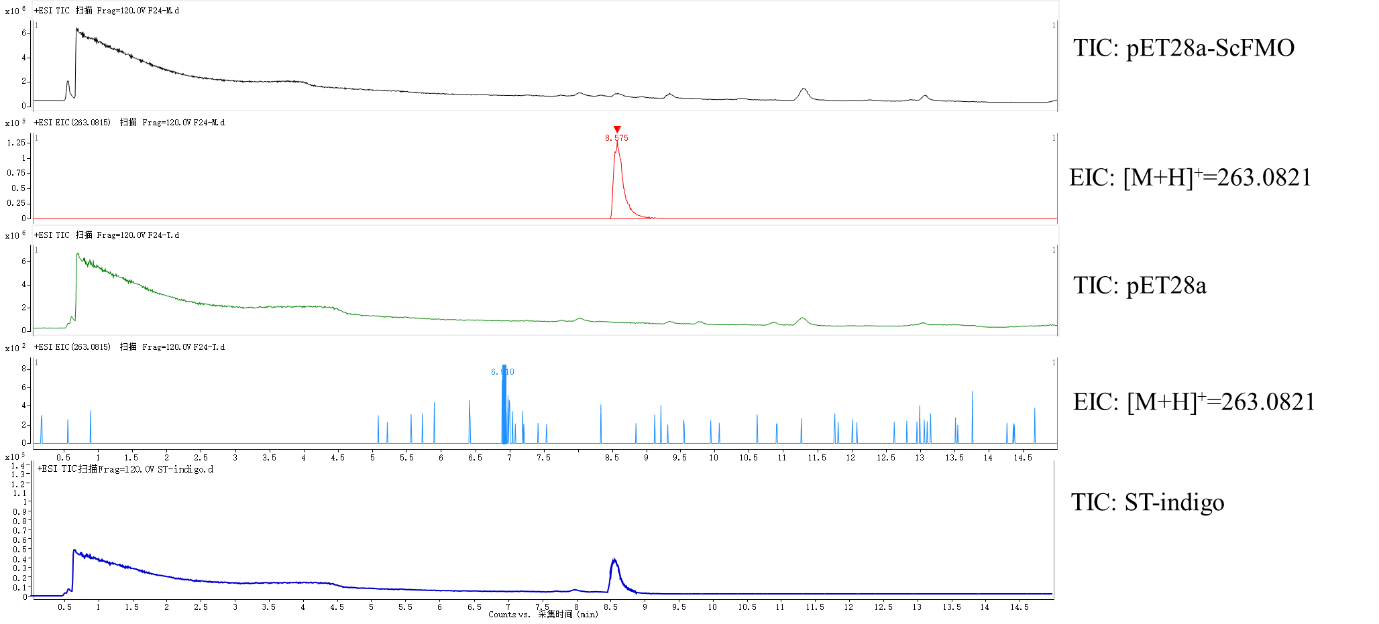


**Figure S4** LC/MS analysis of the indigo production reaction.


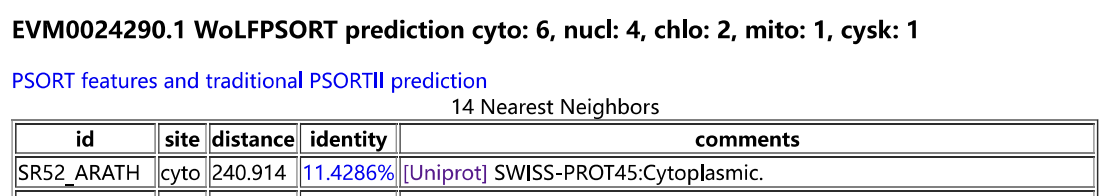


**Figure S5 Subcellular localization site prediction for ScFMO1**


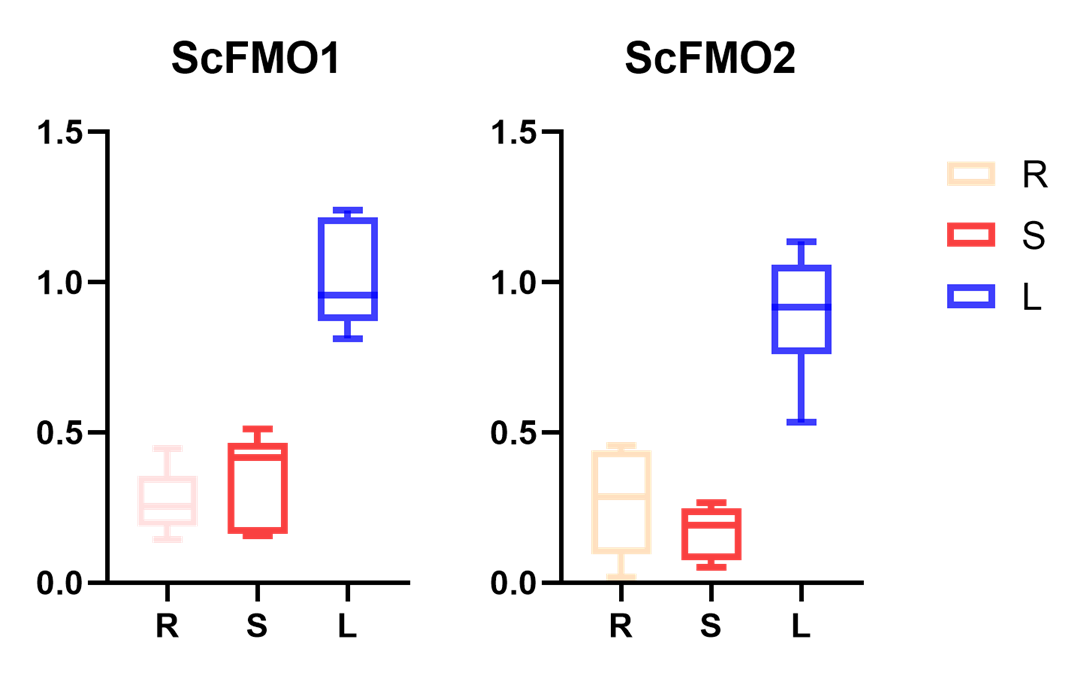


**Figure S6** **qRT-PCR validation of ScFMO1 and ScFMO2**


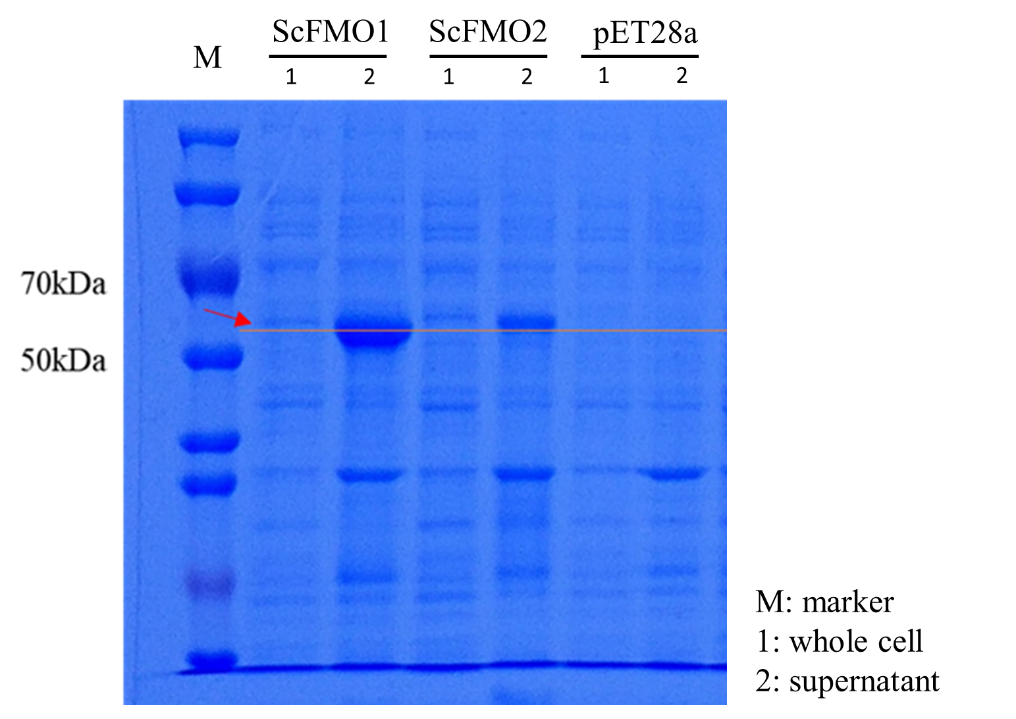


**Figure S7 The SDS-PAGE gel of ScFMO1 and ScFMO2 expressions in *E. coli***


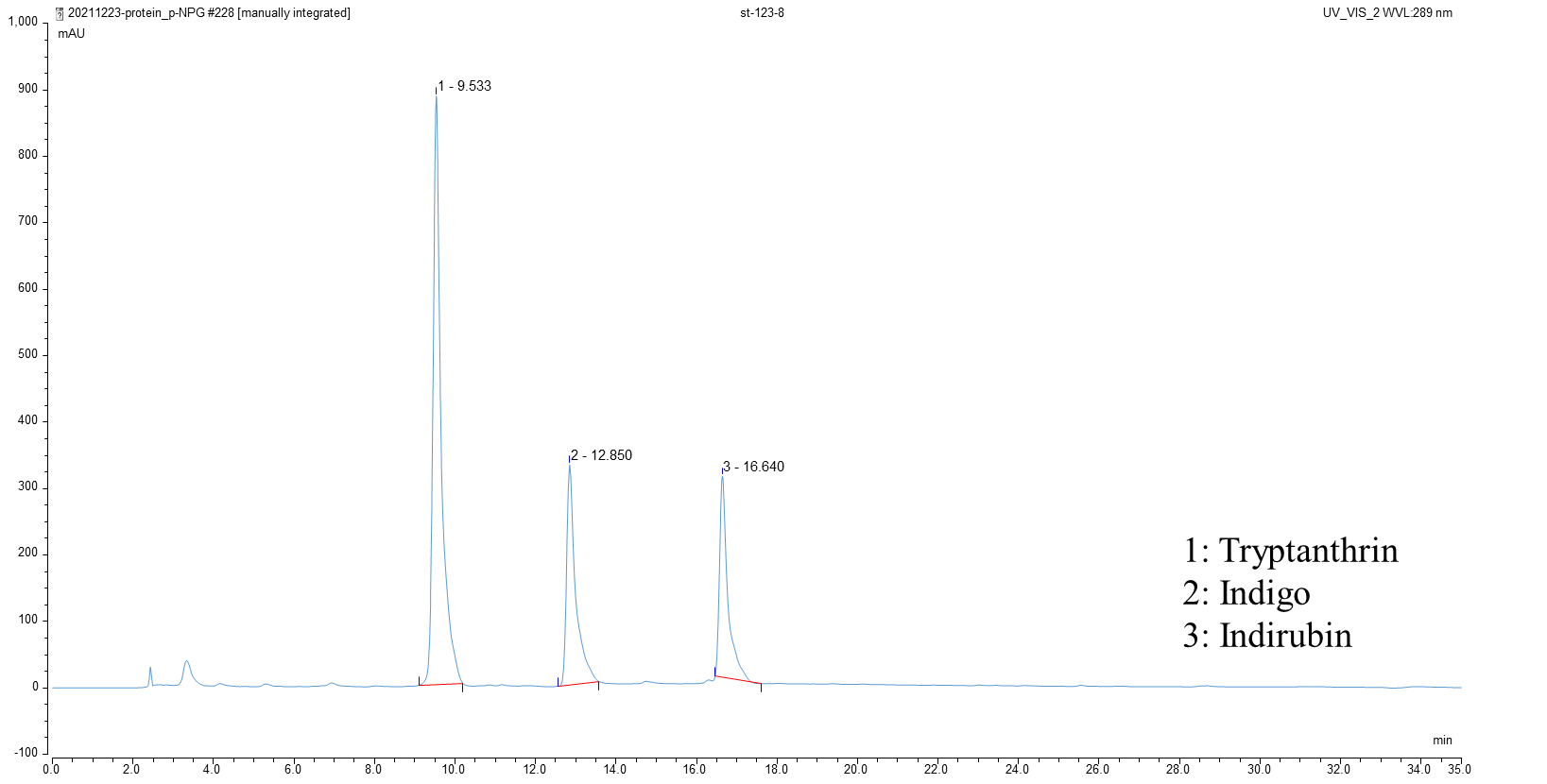


**Figure S8** **The gradient elution chromatography of metabolic analysis for Standards**
